# An acetyltransferase controls the metabolic flux in rubromycin polyketide biosynthesis by direct modulation of redox tailoring enzymes†

**DOI:** 10.1039/d2sc01952c

**Published:** 2022-05-17

**Authors:** Marina Toplak, Adelheid Nagel, Britta Frensch, Thorsten Lechtenberg, Robin Teufel

**Affiliations:** Faculty of Biology, University of Freiburg Schänzlestrasse 1 79104 Freiburg Germany; Pharmaceutical Biology, Department of Pharmaceutical Sciences, University of Basel Klingelbergstrasse 50 4056 Basel Switzerland robin.teufel@unibas.ch

## Abstract

The often complex control of bacterial natural product biosynthesis typically involves global and pathway-specific transcriptional regulators of gene expression, which often limits the yield of bioactive compounds under laboratory conditions. However, little is known about regulation mechanisms on the enzymatic level. Here, we report a novel regulatory principle for natural products involving a dedicated acetyltransferase, which modifies a redox-tailoring enzyme and thereby enables pathway furcation and alternating pharmacophore assembly in rubromycin polyketide biosynthesis. The rubromycins such as griseorhodin (*grh*) A are complex bioactive aromatic polyketides from Actinobacteria with a hallmark bisbenzannulated [5,6]-spiroketal pharmacophore that is mainly installed by two flavoprotein monooxygenases. First, GrhO5 converts the advanced precursor collinone into the [6,6]-spiroketal containing dihydrolenticulone, before GrhO6 effectuates a ring contraction to afford the [5,6]-spiroketal. Our results show that pharmacophore assembly in addition involves the acetyl-CoA-dependent acetyltransferase GrhJ that activates GrhO6 to allow the rapid generation and release of its labile product, which is subsequently sequestered by ketoreductase GrhO10 and converted into a stable intermediate. Consequently, the biosynthesis is directed to the generation of canonical rubromycins, while the alternative spontaneous [5,6]-spiroketal hydrolysis to a ring-opened pathway product is thwarted. Presumably, this allows the bacteria to rapidly adjust the biosynthesis of functionally distinct secondary metabolites depending on nutrient and precursor (*i.e.* acetyl-CoA) availability. Our study thus illustrates how natural product biosynthesis can be enzymatically regulated and provides new perspectives for the improvement of *in vitro* enzyme activities and natural product titers *via* biotechnological approaches.

## Abstract 2

Characterization of the acetyltransferase GrhJ reveals the surprising acetylation of flavoenzyme GrhO6 in rubromycin polyketide biosynthesis, showcasing a novel principle for the enzymatic regulation of secondary metabolic pathways.

## Graphical abstract

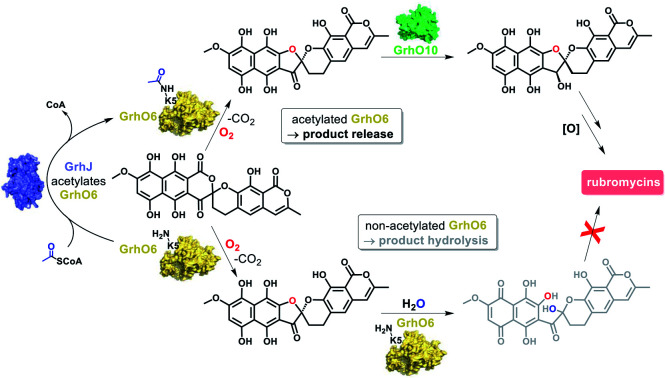

## Introduction

Aromatic polyketides produced by type II polyketide synthases (PKSs) are a well-studied and pharmacologically important group of natural products from Actinobacteria.^1,2^ Due to their potent bioactivities, some of these compounds are in clinical use, *e.g.*, the broad-spectrum antibiotic tetracyclin or the anti-tumorigenic anthracyclins.^2^ Among the most complex and highly oxidized aromatic polyketides are the members of the rubromycin family, which have notable antimicrobial activities as well as cytotoxic effects on cancer cell lines.^1,3–7^ Furthermore, rubromycins have been suggested as potential lead structures for drug development, as they inhibit proteins of medical interest such as human telomerase, HIV reverse transcriptase, or DNA helicase.^8,9^ Due to their complex chemical nature, resulting from the unique [5,6]-spiroketal-group linking coumarin and naphthazarin moieties, the chemical synthesis of rubromycin-type polyketides such as griseorhodin A (1) or hyaluromycin (2) remains challenging,^1^ making it worth considering the development of combined organo-enzymatic synthesis strategies. Also for that reason, the biosynthetic pathways leading to the mature rubromycin polyketides require detailed investigation.

**Fig. 1:**
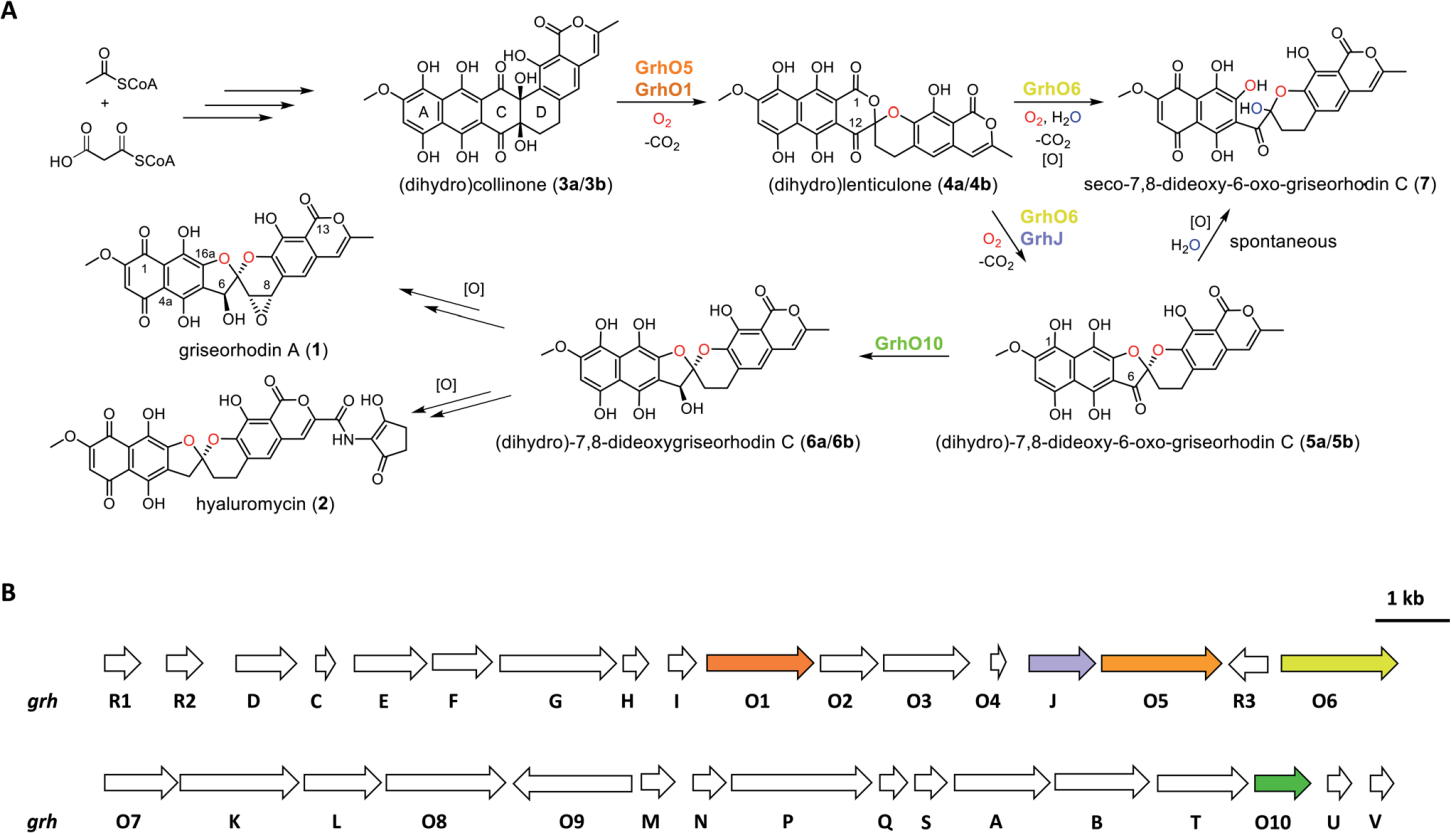
Simplified schematic representation of the biosynthesis pathways of the rubromycin polyketide family leading to mature 1 and 2 (A) and depiction of the 1 biosynthetic gene cluster (B). (A) Overview of the key reaction steps leading from acetyl-CoA and malonyl-CoA to 1 and 2. The roles of GrhJ and GrhO10 were elucidated in this work. Oxygen atoms incorporated by enzymatic oxygenation and spontaneous hydrolysis are colored in red and blue, respectively. The on-pathway steps most likely proceed *via* ring A-reduced hydroquinonic intermediates (compounds denoted with a), while autooxidation [O] products are often observed after compound preparation and isolation (compounds denoted as b). Note the different carbon numbering of 4a/4b compared to 1, 2, 5a/5b, and 6a/6b, which was maintained according to previous reports and is based on the numbering of the oxidized (b) compound versions. (B) Graphical representation of the griseorhodin A (*grh*) biosynthesis gene cluster (shown in two rows; *grhO6* and *grhO7* are adjacent in the genome) with the genes coding for the enzymes relevant to this work colored in accordance with the reaction scheme shown in panel (A).

The identification and investigation of the griseorhodin A biosynthetic gene cluster (BGC) in *Streptomyces* sp. JP95 revealed that early steps in the formation of rubromycin-type polyketides involve a minimal type II PKS, ketoreductases, cyclases/aromatases as well as diverse tailoring enzymes (Fig. 1).^4,6^ Notably, similar to many other pharmacophores of aromatic polyketides,^10–14^ formation of the hallmark bisbenzannulated spiroketal during late stage biosynthesis was attributed to distinct flavin-dependent oxidoreductases based on gene knock out experiments and metabolic studies.^5^ Recently, the biosynthesis of the spiroketal could be elucidated in more detail by *in vitro* studies with enzymes encoded by the 1 BGC (*grh*).^15,16^ Notably, these biosynthetic steps most likely proceed *via* ring A-reduced hydroquinonic intermediates (compounds introduced below denoted with a), while *ortho*-quinonic intermediates and final pathway products that are typically described in the literature are the result of autooxidation (compounds denoted as b).^16^ Accordingly, the advanced pentangular pathway intermediate dihydrocollinone (3a) serves as a precursor for the generation of the [6,6]-spiroketal intermediate dihydrolenticulone (4a), which is subsequently converted to dihydro-7,8-dideoxy-6-oxo-griseorhodin C (5a) that features the mature [5,6]-spiroketal motif. While 3a and 4a are moderately stable under aerobic conditions and can be detected by liquid chromatography (LC) methods, compounds featuring a mature pharmacophore such as 5a autooxidize too rapidly for detection. The formation of the spiroketal is facilitated by several flavoenzymes, which are known to be mechanistically versatile.^10,11,13,17–25^ Accordingly, generation of 4a mainly depends on the group A flavoprotein monooxygenase (FPMO) GrhO5,^15^ which is assisted by flavoprotein oxidase GrhO1.^16^ The subsequent conversion of the [6,6]-spiroketal-containing 4a into 5a*via* oxygenation and succeeding decarboxylative ring contraction depends on a second group A FPMO, GrhO6, while further downstream steps require further investigation and presumably proceed *via* dihydro-7,8-dideoxygriseorhodin C (6a) to the mature rubromycins such as 1 and 2.^16^ Both FPMOs were furthermore shown to moonlight as ring A reductases to increase substrate reactivity and likely enable salvaging of intracellular autooxidation products such as 3b and 4b.^15,16^ Notably, the GrhO6 product 5a/5b could not be directly observed previously but rather seco-7,8-dideoxy-6-oxo-griseorhodin C (7), which was shown to arise from the spontaneous hydrolysis of ring C of unstable 5a (presumably driven by the ring strain caused by the sp^2^-hybridized C6-ketone) and ring A autooxidation.

Open questions remain, *inter alia*, regarding the reduction of the C6-ketone of 5a/5b to 6a/6b*en route* to the mature rubromycins that may be catalyzed by the putative ketoreductase GrhO10 and the corresponding functional homologs such as HyalO10 from 2 biosynthesis.^5^ In addition, the role of GrhJ/HyalJ, which belong to the acetyltransferase subfamily of GCN5-related *N*-acetyltransferases (GNATs), is particularly intriguing. Previously, investigation of the Δ*grhJ* and Δ*grhO6* deletion mutants of the heterologous producer *S. albus* J1074 showed that 1 formation was abolished in both strains, which instead accumulated 4b as one of the major compounds. Accordingly, it was suggested that both enzymes play a role in [5,6]-spiroketal biosynthesis and the conversion of 4b into 5b. However, more recent studies showed that GrhO6 alone seemed sufficient for the *in vitro* formation of 5a/5b, although only 7 could be directly detected in adequate amounts (Fig. 1),^16^ raising the question as to how the rubromycin-producing bacteria prevent formation of this shunt product *in vivo*. In this work, we investigated the late stage redox tailoring of rubromycin polyketides and interrogated the cryptic role of the involved acetyltransferases, which revealed an unanticipated novel principle for controlling the metabolic flux in polyketide biosynthesis *via* direct acetylation and activation of key tailoring enzyme GrhO6.

## Results

### Unstable (dihydro-)7,8-dideoxy-6-oxo-griseorhodin C (5a/5b) is the substrate of the ketoreductase GrhO10

**Fig. 2:**
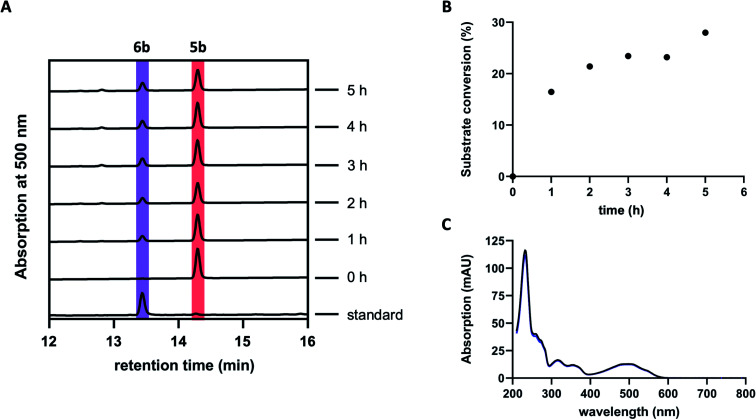
Conversion of isolated 5b by the ketoreductase GrhO10. (A) Chromatograms showing the time-dependent conversion of 5b (dark red) into 6b (purple) by GrhO10. (B) Time-course graph for the conversion of 5b into 6b. (C) Comparison of the virtually identical UV-visible absorption spectra of pure standard 6b (black line) and of the GrhO10 product (blue line).

Previous studies suggested GrhO10 as the most likely candidate for the envisaged C6-ketone reduction of 5.^26^ To investigate this, GrhO10 was heterologously produced in *E. coli* and purified by Ni-NTA chromatography, yielding highly pure and concentrated recombinant protein based on SDS-PAGE analysis (Fig. S1 and S2†). The *in vitro* activity of GrhO10 was then examined by *in situ* production of its putative substrate 5 from 3b*via* a reaction cascade containing NADPH, GrhO5, GrhO1, and GrhO6. However, the GrhO10-mediated 6a/6b formation could not be observed; instead, only compound 7 accumulated.^16^ Further scrutiny of the coupled assays revealed that the seemingly complete spontaneous hydrolysis of GrhO6-formed 5a/5b into 7 most likely prevented 6a/6b formation by depriving GrhO10 of its substrate (Fig. S3†). Therefore, 5b was produced by chemical oxidation of the C6-hydroxyl group of the more stable 6b to investigate the role of GrhO10. For this purpose, 6b was first obtained from the mutant strain *S. albus* J1074 KR7 and oxidized to 5b by Dess–Martin periodinane as previously reported.^16^ Despite the low solubility of 5b, limiting the availability of this compound, the enzymatic formation of 6b was clearly observed in the presence of GrhO10 (Fig. 2), whereas in its absence only 7 was formed (Fig. S4†).

Hence, these data confirm that GrhO10 represents a ketoreductase (KR) and acts on 5a/5b rather than 7. A closer inspection of GrhO10 as well as the functional homologues HyalO10 and RubG (rubromycin biosynthesis) revealed high similarity of these enzymes to the structurally characterized C17/C19 ketoreductase ARX21 from arixanthomycin biosynthesis (60% identity, 100% coverage).^26^ Multiple sequence alignment and homology modeling indicated that all important catalytic and substrate binding residues identified in ARX21 (S144, Y157, K161 and Y210, R154, respectively) are conserved in GrhO10 and its homologs (Fig. S5 and S6†), which is in line with the fact that they also act as C17 KRs on substrates with comparable chemical structure.

### A dedicated acetyltransferase is instrumental to (dihydro-)7,8-dideoxy-6-oxo-griseorhodin C (5a/5b) formation

**Fig. 3:**
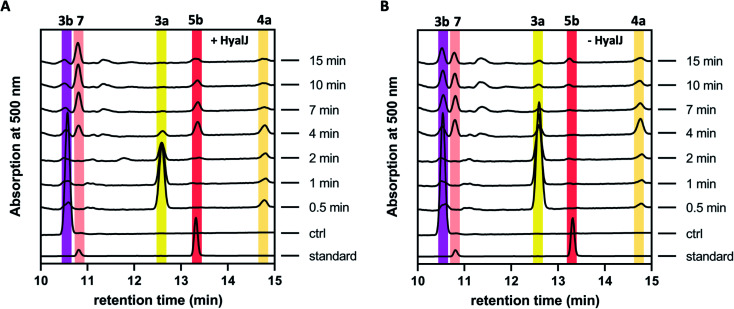
HPLC-chromatograms showing the time course for the formation of 5b in a reaction cascade containing 3b, NADPH, GrhO5, GrhO1, GrhO6 ± HyalJ. (A) In the reaction with HyalJ, after the initial reduction of 3b (ctrl, pink line – 10.5 min) to 3a (yellow line – 12.5 min) by GrhO5, 4a (pale yellow line – 14.7 min) is formed by the combined action of GrhO5 and GrhO1. Compound 4a is then further converted into 5a/5b (dark red line – 13.3 min) by GrhO6 together with HyalJ. At the same time, the hydrolysis product 7 (light red line – 10.7 min) is generated from 5a/5b. (B) In the reaction without HyalJ, the produced 4a is further converted into 7 (light red line – 10.7 min) by GrhO6 without evident accumulation of 5a/5b. In this case, 5a/5b (standard, dark red line – 13.3 min) could not be detected. Note, the small peak appearing at around 13.1 min after about 10 min reaction time based on the UV-visible absorption characteristics does not match 6a/6b. Instead, it is a minor side product mostly observed in enzyme assays lacking GrhO6 (see Fig. S11†).

The fact that enzyme assays containing 3b, NADPH, GrhO5, GrhO1, GrhO6 and GrhO10 did not yield any 5a/5b further raised the question, whether an additional enzyme would be required for the generation of this compound *in vivo*. As the previous gene knock out studies suggested a possible involvement of the putative acetyltransferase GrhJ, this enzyme was heterologously produced in *E. coli* and purified by affinity chromatography. However, his-tagged GrhJ turned out to be mostly insoluble whereas MBP-tagged GrhJ was unstable and underwent spontaneous degradation (Fig. S7 and S8†). In contrast, HyalJ, a predicted functional homolog of GrhJ from 2 biosynthesis, could be obtained as soluble and stable protein with an N-terminal GB1 solubility-tag (Fig. S9†). Gratifyingly, when HyalJ was added to the reaction mixture with 3b, NADPH, GrhO5, GrhO1, and GrhO6, a new peak was observed after 2–4 min incubation time, which was identified as 5b by comparison with the chemically prepared standard (Fig. 3 and S10–12†).

These results confirmed a cryptic role for the GNATs in promoting 5a/5b production and/or in counteracting spontaneous ring C hydrolysis to 7. Compound 7 was only recently identified and shown to be produced to some extent *in vivo* in different rubromycin polyketide producer strains (Fig. S13†).^16^ However, based on our results, an increased formation of 7 would be expected in the Δ*grhJ* strain knockout out strain *S. albus* J1074 KR42,^5^ which was originally reported to accumulate 4a(b) as major product.^16^ Gratifyingly, the cultivation and re-analysis of this strain indeed showed that 7 is the most dominant polyketide-related compound *in vivo* alongside 4b, fully consistent with the *in vitro* results (Fig. S14†). However, although 5a/5b was faster formed and clearly accumulated in the enzyme assays, its spontaneous hydrolysis also proceeded at significantly higher rates compared to pure 5b in the same buffer without enzymes (Fig. 3, S4 and S10†). Further scrutiny of the assays revealed that reduced 5a is substantially more susceptible to undergo hydrolysis than its oxidized counterpart 5b. Therefore, GrhO5 and GrhO6 are likely responsible for the accelerated 5a/5b decomposition due to their ring A reductase activities.^15,16^

Because GrhJ and HyalJ are predicted GNATs, acetyl-CoA (AcCoA) was next added to assay mixtures containing 3b, NADPH, GrhO5, GrhO1, GrhO6 together with HyalJ. Interestingly, supplementation of AcCoA resulted in an increase in 5a/5b formation *in vitro* (Fig. S15 and Table S1†), which, surprisingly, became more pronounced with each freezing–thawing cycle that HyalJ had undergone before (Fig. S16†). Therefore, we speculated that HyalJ is purified from *E. coli* as holo-enzyme with bound AcCoA, but over time loses its ability to boost 5a/5b formation due to the degradation of AcCoA. To test this hypothesis, the enzyme was freshly produced, purified and immediately sacrificed by the addition of 1 eq. (v/v) EtOAc + 10% FA. Then, the organic phase was removed, and the aqueous layer analyzed by UPLC-HRMS, which clearly confirmed the presence of AcCoA (Fig. S17 and S18†). Interestingly though, UPLC-MS analysis revealed that HyalJ is not only co-purified with AcCoA, but also with almost identical amounts of propionyl-CoA and methylmalonyl-CoA as well as minor amounts of malonyl-CoA (Fig. S19–S22†). To find out, whether binding of these acyl-CoA derivatives also promotes 5a/5b formation, enzymatic assays with apo HyalJ (which has virtually no effect on 5a/5b formation in the absence of free AcCoA) were repeated in the presence of supplemented propionyl-CoA, malonyl-CoA and methylmalonyl-CoA. While propionyl-CoA and methylmalonyl-CoA had an equal effect to AcCoA, the addition of malonyl-CoA to the reaction mixture did not result in increased 5a/5b formation, consistent with the minuscule amounts of malonyl-CoA co-purified with HyalJ compared to the other CoA-derivatives (Fig. S19 and S23†).

### HyalJ directly activates GrhO6 by ac(et)ylation

Having demonstrated that acyl-CoA esters are crucial for the enzymatic activity of HyalJ, still the question of its mode of action remained. GNATs generally acetylate amino groups of metabolites or proteins. In the latter case, the modification often occurs at the ε-amino groups of internal lysine side chains, which, *e.g.*, results in their stabilization or (in)activation.^27–29^ Rubromycins and their precursors lack amino groups and acetylated intermediates were expectedly not observed. Moreover, separate assays showed that purified 7 cannot be converted into 5 by HyalJ in the presence of AcCoA, thus ruling out the unlikely scenario that the hydroxyl group at the former spiro-carbon of 7 is transiently acetylated to promote ring closure to 5 (Fig. S24†). Hence, the most plausible explanation for the obtained results appeared that HyalJ directly modifies GrhO6. To further test the envisaged mode of action, putative acetylation-sites in GrhO6 were first identified by bioinformatic predictions,^30–32^ which implied two Lys residues (K5, K57) and to a lesser extent K425 close to the C-terminus. To find out whether the acetylation of any of these Lys residues determines the enzymatic activity of GrhO6, three variants were generated, in which either one of the Lys was replaced by a methionine (K5M, K57M, K425M). Recombinant production and purification of the three variants yielded similar amounts of pure protein compared to the wild type enzyme only for GrhO6-K57M, whereas GrhO6-K425M was less soluble with weakly bound FAD-cofactor and GrhO6-K5M was not produced at all (Fig. S25 and S26†). Nevertheless, both the K57M – as well as the K425M variant exhibited similar enzymatic activities like the wild type protein and both were able to mediate the generation of 5a/5b in the presence of HyalJ (Fig. S27†). The fact that the K5M-variant was not even overproduced, in contrast, indicates that K5 is an important residue in GrhO6.

**Fig. 4:**
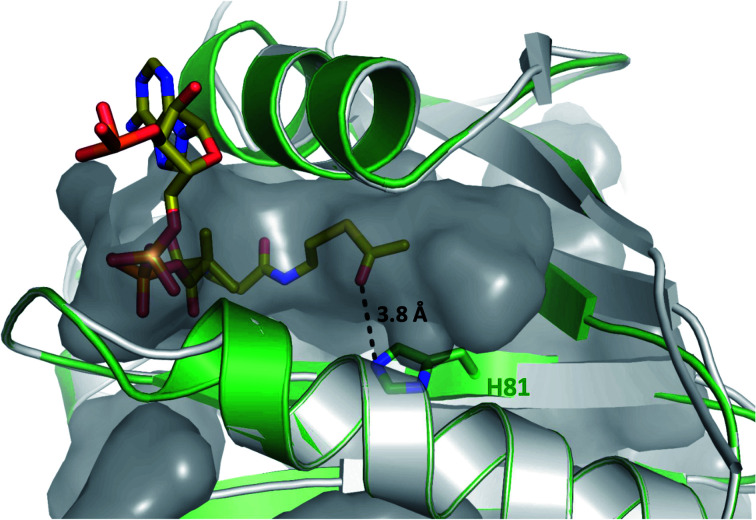
Close-up view of the putative active site of a HyalJ model with the AcCoA binding pocket displayed in surface mode. The fact that the CoA binding site is likely much larger than required for AcCoA binding, is in line with the observation that HyalJ also binds the bulkier CoA-derivatives propionyl-CoA, malonyl-CoA and methylmalonyl-CoA.

To further investigate whether K5 is the site of acetylation, freshly purified GrhO6 (as control) and GrhO6 after turnover of 4b in the presence of AcCoA and HyalJ were in-gel digested with an *N*-Asp protease and analyzed by UPLC-HRMS. Despite low signal intensity of the peptide of interest due to unfavorable protease cleavage sites and poor ionization, analysis of the extracted protein fragments indicated that reacted GrhO6 is acetylated at K5 (Fig. S28†), fully in line with the bioinformatic predictions. Accordingly, a [M + 2H]^2+^-fragment with 332.661 *m*/*z* corresponding to DTK(Ac)GTT was detectable that was absent in the control. At the same time, the unmodified counterpart ([M + 2H]^2+^, 311.656 *m*/*z*) of this fragment was exclusively detected in the control. These results corroborate that the activation of GrhO6, which boosts 5a/5b formation, depends on the covalent modification of K5 by the ac(et)yltransferase HyalJ. GNATs typically have a strictly conserved fold, ideally suited for homology modeling.^27,33,34^ Accordingly, the predicted structure of HyalJ revealed the putative AcCoA binding site as well as catalytic residues. Based on its position, His81 may thus function as critical residue of the oxyanion hole for stabilization of the negative charge in the transition state following the attack of GrhO6's K5 side chain on the thioester bond of the CoA-ester substrates. The active site pocket itself is sizeable, likely explaining why HyalJ can also bind and activate bulkier CoA-esters such as propionyl-CoA and methylmalonyl-CoA (Fig. 4 and S29†).

### Acetylated GrhO6 facilitates (dihydro-)7,8-dideoxy-6-oxo-griseorhodin C (5a/5b) turnover into (dihydro-)7,8-dideoxygriseorhodin C (6a/6b)

As a result of the improved *in vitro* formation of 5a/5b enabled by the HyalJ-mediated activation of GrhO6, GrhO10 was again added to the reaction cascade with 3, NADPH, GrhO5, GrhO1, GrhO6 and HyalJ to test for the anticipated reaction product 6a/6b. Indeed, the reduction of the C6-ketone group of 5a/5b by GrhO10 was successful this time; however, the turnover efficiency was low, resulting in a high degree of hydrolysis product 7 aside from enzymatically produced 6a/6b (Fig. S30, panel A, red line†). Since GrhO10 showed a high tendency toward aggregation and precipitation under assay conditions, its homolog from the hyaluromycin gene cluster (HyalO10) was heterologously produced and purified (Fig. S1 and S2†). When GrhO10 was substituted for HyalO10 in the enzymatic assays, 6a/6b was formed in much higher quantities (Fig. S30†). Interestingly though, in the reaction mixtures with HyalO10, a second new peak (8) was observed (also detected with GrhO10, but less pronounced due to the low overall turnover efficiency), which exhibited UV-vis absorption characteristics similar to oxidized lenticulone (4b) (Fig. S31†). To characterize this compound in more detail, the isolated peak was subjected to UPLC-HRMS/MS analysis, which revealed a parent mass of 521.073 ([M − H]^−^) and fragments of 317.030 and 275.019 *m*/*z* (Fig. S32†). These data indicated that the ketoreductases are also capable of catalyzing keto reduction of 4a/4b, most likely at C12 of ring C that represents the equivalent position to C6 of 5a/5b. This is further supported by the observation that 8 accumulated in the presence of HyalO10 when GrhO6 was absent (Fig. S33†). Further assays in which 8 was produced *in situ* prior to the addition of GrhO6, confirmed that this compound cannot be further converted and thus represents a shunt product (Fig. S34†). In addition, substantial amounts of 4a(b) but hardly any 7 were found in these assay mixtures, suggesting that 8 serves as inhibitor for the monooxygenase GrhO6. To confirm this, *in situ* produced 8 and 4a(b) were mixed with pure GrhO6. As expected, GrhO6 showed strongly decreased activity on 4a(b) even in the presence of only small amounts of 8 (Fig. S35†). Overall, these results establish that the GNATs are obligatory in late stage tailoring of rubromycin polyketides to generate an ac(et)ylated form of GrhO6 and thereby enable assembly of a stable [5,6]-spiroketal with the help of GrhO10.

## Discussion

In our study we could expose a novel principle for controlling the fate of advanced pathway intermediates and the formation of final products by direct modulation of redox tailoring enzymes in aromatic polyketide biosynthesis. Previously, the reversible acylation of enzymes from central metabolism was proposed to allow bacteria to rapidly respond to environmental changes.^35^ Moreover, total intracellular acetylation levels have been reported to affect natural product biosynthesis, *e.g.*, in the case of the polyketide erythromycin from *S. erythraea*. However, identified acetylated enzyme targets were involved in precursor supply rather than direct biosynthetic steps.^36^ To the best of our knowledge, our findings present the first case in which dedicated ATs directly interfere with tailoring enzymes to control the metabolic flux in bacterial natural product biosynthesis. Interestingly, these enzymes are encoded by all currently known rubromycin BGCs (for production of 1, 2, and heliquinomycin) and based on blastp-searches also seem to be present in (α/β/γ)-rubromycin-producing *S. collinus*^16^ (whose BGC was only partially sequenced), suggesting a universal role in controlling rubromycin polyketide biosynthesis.

**Fig. 5:**
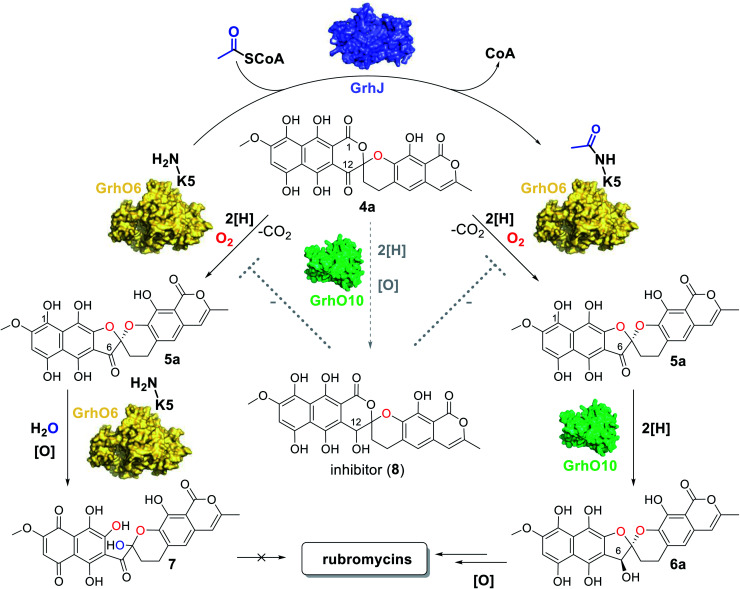
Overview of the reactions catalyzed by the monooxygenase GrhO6, the acetyltransferase GrhJ and the ketoreductase GrhO10 in the late steps of rubromycin polyketide biosynthesis. In addition, the effect of inhibitor 8 on the conversion of 4a into 5a by GrhO6 is indicated. See text for details.

As malonyl-CoA, the main precursor for rubromycin biosynthesis, is directly formed from acetyl-CoA, it stands to reason that the GNATs monitor the availability of pathway precursors. Accordingly, a lack of acetyl-CoA (due to an arrest of the citric acid cycle), indicative of a phase of starvation, may prompt a rapid halt of rubromycin biosynthesis and thereby prevent stagnation or even cell death from nutrient depletion, while at the same time freeing resources, *e.g.*, for sporulation.^37^ In contrast, during high-nutrient conditions, rubromycin polyketide biosynthesis is affordable and pushed forward by acetylated GrhO6. Notably, the metabolic profile observed for the Δ*grhJ* knock-out strain of the heterologous producer most likely mimics that of the starved-out cell. It is therefore tempting to speculate that the respective final products in each scenario (4a/4b and 7 as opposed to mature rubromycins during starvation and high nutrient conditions, respectively) fulfill different biological functions and thus enable a broader metabolic adaptability and thus increased survivability. For example, 4a/4b has antibacterial as well as antiproliferative activity and was furthermore shown to act as inhibitor of the serine protease human leukocyte elastase,^5^ clearly highlighting the bioactivity of this compound. Evidently, the impaired functionality of non-acetylated GrhO6 leads to accumulation of 7 and prevents 6a/6b formation. Concurrently, 4a/4b can be partially transformed into 8 by the action of GrhO10/Hyal010, as verified by the *in vitro* assays. The potent inhibitor 8 then further impedes GrhO6 functionality, which may explain why the cells of the Δ*grhJ* knock-out strain do not completely convert 4a/4b into 7 and instead accumulate both compounds (Fig. 5). These data, however, are also a reminder that metabolic profiles of bacteria strongly depend on the growth conditions, as nutrient-rich media in the laboratory typically do not reflect the natural environment, in which nutrient scarcity may often be the rule rather than the exception. So far, it remains unclear if GrhO6 remains activated for its entire lifetime or whether deacetylases exist that target the enzyme; obvious enzyme candidates are not encoded in the rubromycin BGCs or their vicinity. Further studies are required to address these open questions and the biological implications of the reported metabolic adjustments.

In the *in vitro* assays, conditions for shunt product 7 formation are favorable due to the presence of NADPH in combination with non-acetylated GrhO6. This leads to a boost in ring A reduction of autooxidized *ortho*-quinonic pathway intermediates and thus to the accumulation of 5a, which is evidently more susceptible to undergo spiroketal hydrolysis compared to 5b. Most likely, this is equally true *in vivo* under the reducing conditions present in the cell, which is supported by the observation that the Δ*grhJ* knock-out strain, which can only produce non-acetylated GrhO6, accumulates major amounts of 7. However, the complete lack of 6a/6b formation in the coupled assays with non-acetylated GrhO6 even in presence of a large excess of GrhO10 is surprising and should at least enable the partial conversion of 5a/5b into stable 6a/6b. A plausible explanation for these observations is that product release by non-acetylated GrhO6 is significantly slowed down, resulting in the hydrolysis of 5a/5b into 7 within its active site and thereby depriving the ketoreductase of its substrate. Hence, it appears likely that GrhJ-mediated acetylation may affect specific interactions of GrhO6 with 5a/5b and/or conformational dynamics required for product release. This is substantiated by the fact that group A FMPOs are well known to undergo conformational changes and, *e.g.*, feature mobile flavin cofactors whose movements are tightly controlled by the protein matrix and depend on the presence of substrates.^10,15,19,20,38–40^ Overall, these results also emphasize that the different redox states of (hydro)quinonic pathway intermediates and final products drastically affect the molecular properties and therefore need to be taken into account with respect to their biosynthesis, stability and activity.

In summary, we could show that the direct modification of a key redox tailoring enzyme by a dedicated *N*-acetyltransferase is required to enable the production of advanced on-pathway precursors *en route* to the rubromycins. Our *in vitro* reconstitution of the biosynthetic steps and their regulation combined with the *in vivo* observations from the Δ*grhJ* knock-out strain clearly confirm that these acetyltransferases govern rubromycin polyketide biosynthesis. Accordingly, this novel principle of controlling the metabolic flux and effectuating pathway branching in polyketide biosynthesis enables the formation of distinct final products with alternate pharmacophores. The results presented herein thus provide deeper insights into the regulation and formation of bioactive secondary metabolites. We anticipate that similar strategies will be exposed in other biosynthetic pathways, which also brings new opportunities for the investigation of enzymes that thus far proved inactive *in vitro* and provides knowledge for future pathway bioengineering efforts as well as for chemoenzymatic approaches for natural product formation.

## Data availability

All relevant data is included in the main text and ESI.†

## Author contributions

A. N. and M. T. performed the cloning, the heterologous production and purification of the proteins and performed the enzyme assays. A. N., M. T., R. T. analyzed and interpreted the results, and generated the figures. A. N. and M. T wrote the Material and method section. B. F. and T. L. characterized the rubromycin polyketide producer strains. T. L. performed initial experiments with GrhO10. M. T. and R. T. wrote the manuscript.

## Conflicts of interest

The authors declare no conflict of interest.

## Supplementary Material

SC-013-D2SC01952C-s001
