# Supplementary material for: An acetyltransferase controls the metabolic flux in rubromycin polyketide biosynthesis by direct modulation of redox tailoring enzymes
Source: Chem Sci. 2022 May 17;13(24):7157–64. doi: 10.1039/d2sc01952c (PMC9215129; doi:10.1039/d2sc01952c)
Supplement: SC-013-D2SC01952C-s001 [file SC-013-D2SC01952C-s001.pdf]

Electronic Supplementary Material (ESI) for Chemical Science.
This journal is © The Royal Society of Chemistry 2022

Supporting Information

An acetyltransferase controls the metabolic flux in rubromycin polyketide biosynthesis by direct modulation of redox tailoring enzymes

Marina Toplak[a], Adelheid Nagel[a], Britta Frensch[a], Thorsten Lechtenberg[a], and Robin Teufel[b]

[a] Faculty of Biology, University of Freiburg, Schänzlestrasse 1, 79104 Freiburg, Germany
[b] Pharmaceutical Biology, Department of Pharmaceutical Sciences, University of Basel, Klingelbergstrasse 50, 4056 Basel, Switzerland

E-mail: robin.teufel@unibas.ch

1. Materials and methods

General

All chemicals and reagents used were obtained from Carl Roth (Karlsruhe, Germany), Sigma-Aldrich (St. Louis, MO, USA), Fisher Scientific (Hampton, NH, USA) and Biomol (Hamburg, Germany). Enzymes and materials used for molecular cloning were purchased from New England Biolabs (NEB), Thermo Fisher Scientific (Waltham, MA, USA), and Qiagen (Hilden, Germany) and oligonucleotides (PCR-primers) were obtained from Sigma Aldrich. For protein purification and concentration, equipment (Ni-NTA-columns, gelfiltration columns) from GE Healthcare/Cytiva (Chicago, IL, USA) and PALL (New York, USA) was used. Agarose- and SDS-PAGE analysis was performed using devices from Bio-Rad (Hercules, CA, USA). *S. albus* strains (pMP31, KR8, KR7 and KR42) were kindly provided by Prof. Dr. Jörn Piel (ETH Zürich) and *A. ianthinogenes* and *S. puniceus* strains were obtained from the DSMZ (Braunschweig, Germany).

Nucleotide sequence of codon-optimized GrhO10

ATGGAAAACCTGTATTTCCAGAGTATGGAACTGGGTCTGCAAGGTAAAAAACTGCTGGTGACCGGTGGCAGCCGTGGTATTGGTCGCG
GCGTTGTGCTGGCCGCAGCCAGAGCAGGCGCAGATGTGCTGACCTGCTATGTTAATGATAGTGAACATGTGGAAAGCCTGCGTCGTGAA
CTGAAAGCACTGGGTGGCGATCATGAAGTTGTGCGTGCAGATGTTGGCGATCTGGATCAGGTTGATGCACTGGTGGGTTGGGCCCGTG
AACGTTATGGTAAACTGGATGGTATTGTTAATAACGCAGGTGTGATTAGTCATATTCCGTTTGCCGAACTGGCACCGGATGAATGGAGTC
GCGTTCTGGATACCAATCTGACCGCAGCATATCGTGTGATTCAGCAGAGTCTGCCGCTGCTGGGCAGCGGTGCCAGTGTGGTTAATATTG
GCAGTCGTGGCGCCGCCGCCGGTATTCCTCTGCGTGCTCATTATACCGCCGCAAAAGCAGCCATGATTGGTCTGACCCGCAGTCTGGCAA
AAGAACTGGGTGGCAAAGGTATTCGTGTTAATGTTGTGGCCCCGGGTGTTATTGAAACCGAAGCATTTGATACCATGCCGGCAGAACGC
GCCGAAGGCCTGCGCGCAATGTATGCACAGAAAACCGCCCTGGCACGCCTGGGTCGTGTTGATGAACTGGCCGCCCCGGTGCTGTTTCT
GCTGAGTGATCTGGCAGCATACGTTACCGGTGAAACCCTGAATGTTGATGGTGGTATT**TAA**

Protein sequence of GrhO10

MENLYFQSMELGLQGKKLLVTGGSRGIGRGVVLAAARAGADVLTCYVNDSEHVESLRRELKALGGDHEVVRADVGDLDQVDA
LVGWARERYGKLDGIVNNAGVISHIPFAELAPDEWSRVLDTNLTAAYRVIQQSLPLLGSGASVVNIGSRGAAAGIPLRAHYTAAK
AAMIGLTRSLAKELGGKGIRVNVVAPGVIETEAFDTMPAERAEGLRAMYAQKTALARLGRVDELAAPVLFLLSDLAAYVTGETL
NVDGGI*

Nucleotide sequence of codon-optimized HyalO10

ATGGAACTGGCCCTGCATGGTAAAAAAGTGCTGATTACCGGCGGTACCCGCGGCATTGGCCGTGGTATTGTGCTGGCAGCAGCACAGG
CAGGCGCAGATGTGCTGACCTGCCATCGTCAGGATGGCGCCGCAGTTGATAGCCTGGTTCAGGCACTGAAAGAAACCGAAGGTGACCA
TCATGTTATTCGCGCAGATGTTGGTGACCTGGATCAGGTTGATCGTCTGGTTAATGAAGCAAAAGATCGTTTTGGTCGCCTGGATGGTGT
GGTGAATAATGCCGGTGTGATTAGCCATGTTCCGTTTGCAGAACTGCCGGCAGCCGAATGGAGCCGTATTCTGGATACCAATCTGACCG
CAAGCTATCGTGTGATTCAGCAGGCCCTGCCGCTGCTGGGTGCCGGTTCAAGTGTTATTAATATTGGTAGCCGTGGCGCAGCCGCCGGT
ATTCCGCTGCGTGCACATTATACCGCAGCAAAAGCAGCACTGATTGGTCTGACCCGCAGCCTGGCAAAAGAACTGGGCCCGCAGGGTAT
TCGCGTGAATGTGGTGGCACCGGGCGTGATTGAAACCGAAGCATTTGATGATATGCCGGCAGATCGCGCCGCCGGCCTGAGAGCAACC
TATGCACAGAAAACCGCCCTGGCACGCCTGGGCACCGTTGATGAACTGGCAGGTCCGGTTCTGTTTCTGCTGAGTGATCTGAGTACCTAT
GTTACCGGCGAAACCGTGAATGTGGATGGCGGTATT**TAA**

Protein sequence of HyalO10

MELALHGKKVLITGGTRGIGRGIVLAAAQAGADVLTCHRQDGAAVDSLVQALKETEGDHHVIRADVGDLDQVDRLVNEAKDR
FGRLDGVVNNAGVISHVPFAELPAAEWSRILDTNLTASYRVIQQALPLLGAGSSVINIGSRGAAAGIPLRAHYTAAKAALIGLTRSL
AKELGPQGIRVNVVAPGVIETEAFDDMPADRAAGLRATYAQKTALARLGTVDELAGPVLFLLSDLSTYVTGETVNVDGGI*

Nucleotide sequence of codon-optimized HyalJ

ATGGATGTGCGTGCACTGGATGGTGACGCCCCGGCAACCATTGCAGTGCTGCTGCCGGGTTTTCGCGAAACCATGCGTCGTGAACTGCC
GGCAGATCCGCCGGTGACCGCAGAACTGCTGGCCCGTCTGCTGCAGCGTCGTCATGGTGCAGATCGTATTCTGCTGGTGGCCTTTGATG
GCGCAGAACCGGCAGGTATTGTTAAACTGGGCCTGGATCTGGCAGATCCGGATGGTCCGGGTCATGGTAGTCTGTGGGTTTTTCCGGGC
TTTCGCCGTCGCGGTTATGGTCGCGCCCTGGTGGATGCCGCACTGGCTGCACTGCGTGAACGCGGCCGTGGTCCGCTGCTGGTGGATGC
ACCGCAGGGCAAAGCAGCCGATGGCTTTGCAGCACTGGTGGGCGCACGTTGCACCGCAGTGAATGCACGCAATCGTCTGGTTCTGCGC
GGTCCGGCACATGCAGCCCTGGGTGCTGCCGCAGCCCGTCCTGTTCCGGGTCATGGCCTGCGCCGCTGGGCAGGTCGTTGTCCTGATGA
TCTGGTTGATAGTTATGCCCGCACCTGGGGTGCACTGGATGCCCGTGTGAATGGTCAGGCCAAAGTTCGTGAACCGACCGTTGAAGATG
TTCGTGCCCGTGAAGCCGAAGCCGAACGCGCAGGTCATCGTCAGTATGTGACCGCAGCAGTGCGCACCGATGGCGATGTTGTGGCATAT
AGTACCCTGTATGTTCGCGCAAGTCCGATGGCCGATACCGGCGAAACCTTTGTTGTTCCGGATCTGCGTCGCCGCAGTCTGGCAACCTGG
GTGAAAGCCGATCTGCTGCTGACCGCAGCCCGCGAAAATGCACATCTGGCAGTTGTTCAGGCATTCAATGAAACCACCAATGCAGCAGT
TATTGCCCTGAATCGTCGCCTGGGCTTTAAAGCAGATAGTCATTGGGCCACCTATGCCCTGGCCGATCTGGAAGGTAGTCCGCGC**TAA**

Protein sequence of HyalJ

MDVRALDGDAPATIAVLLPGFRETMRRELPADPPVTAELLARLLQRRHGADRILLVAFDGAEPAGIVKLGLDLADPDGPGHGSL
WVFPGFRRRGYGRALVDAALAALRERGRGPLLVDAPQGKAADGFAALVGARCTAVNARNRLVLRGPAHAALGAAAARPVPG
HGLRRWAGRCPDDLVDSYARTWGALDARVNGQAKVREPTVEDVRAREAEAERAGHRQYVTAAVRTDGDVVAYSTLYVRASP
MADTGETFVVPDLRRRSLATWVKADLLLTAARENAHLAVVQAFNETTNAAVIALNRRLGFKADSHWATYALADLEGSPR*

Cloning and recombinant production of GrhO10

To allow for the recombinant production of GrhO10, the respective gene was purchased from BioCat, codon optimized for *Escherichia. coli* and subcloned into the expression vector pET16b. Upon arrival, the plasmid DNA was dissolved in ddH_2O to a final concentration of 100 ng µL^{-1} and transformed into *E. coli* BL21 (DE3) cells. For protein production, TB-medium supplemented with 100 µg mL^{-1} ampicillin was inoculated with a pre-culture grown in LB-medium containing an equal amount of antibiotic. Cultures were incubated at 37 °C and 130 rpm until and optical density at 600 nm (OD_{600}) of ca. 0.5 was reached. Then, the temperature in the incubator was set to 18 °C and at an OD_{600} of 0.7-0.8 protein production was induced with 0.1 mM IPTG. To maximize the protein yield, cultures were incubated at 18 °C and 130 rpm overnight. The following day, cells were harvested by centrifugation (4 000 g for 15 min) and used for protein purification immediately.

Cloning and recombinant production HyalO10

To allow for the recombinant production of HyalO10, the respective gene was obtained from BioCat, codon optimized for *Escherichia coli*, flanked with NcoI (3') and NotI (5') restriction sites. After restriction digestion, the gene was cloned into the pETM11-His-TEV vector, in frame with the coding sequence for an N-terminal hexahistidine tag. Having confirmed the proper insertion of the gene into the vector by automated sequencing, the recombinant plasmid was transformed into *E. coli* BL21 (DE)-cells for subsequent gene expression. For protein production, TB-medium supplemented with 50 µg mL^{-1} kanamycin was inoculated with a pre-culture grown in LB-medium containing an equal amount of antibiotic. Cultures were incubated at 37 °C and 130 rpm until and optical density at 600 nm (OD_{600}) of ca. 0.5 was reached. Then, the temperature in the incubator was set to 18 °C and at an OD_{600} of 0.7-0.8 protein production was induced with 0.1 mM IPTG. To maximize the protein yield, cultures were incubated at 18 °C and 130 rpm overnight. The following day, cells were harvested by centrifugation (4 000 g for 15 min) and used for protein purification immediately.

Cloning and recombinant production MBP-GrhJ

To allow for the recombinant production of GrhJ as fusion protein with MBP (as his-tagged protein the enzyme is not soluble at all), the respective gene was amplified from isolated cosmid DNA using the following primers - fwd primer: 5ˈ - CCGGGCCATGGTGAGCCTCGAACTG – 3; rev. primer: 5ˈ - CCGGTCCCTGCAGTCACGCCTTGACGG - 3ˈ. After restriction digestion with NcoI (5') and PstI (3'), the gene was ligated into a pMAL-vector linearized with the same enzymes and proper insertion was confirmed by automated sequencing. For protein production, TB-medium containing ampicillin (100 µg mL^{-1}) was inoculated with a pre-culture grown in LB-medium supplemented with the same amount of antibiotic. Cultures were incubated at 37 °C and 130 rpm until an OD_{600} of 0.6-0.7 was reached. Then, the temperature in the incubator was set to 18 °C and protein production was induced with 0.25 mM IPTG. To optimize the protein yield, cultures were incubated at 18 °C overnight, before harvesting the cells by centrifugation (4000 g for 15 min).

Cloning and recombinant production GB1-HyalJ

To allow for the recombinant production of GB1-tagged HyalJ, *hyalJ* was obtained from BioCat, codon optimized for *E. coli* and flanked with NcoI (5') and NotI (3') restriction sites. After restriction digestion, the gene was inserted into a pETM11-His-GB1-TEV vector, in frame with the coding sequences for an N-terminal hexahistidine tag and the solubility enhancer protein GB1 (B domain of protein G). Proper insertion of *hyalJ* was confirmed by automated sequencing and recombinant plasmids were transformed into *E. coli* BL21 (DE3) RP cells. For protein production, TB-medium supplemented with 50 µg mL^{-1} kanamycin and 20 µg mL^{-1} chloramphenicol was inoculated with a pre-culture grown in LB-medium containing an equal amount of antibiotic. Cultures were incubated at 37 °C and 130 rpm until and optical density at 600 nm (OD_{600}) of ca. 0.5 was reached. Then, the temperature in the incubator was set to 18 °C and at an OD_{600} of 0.7-0.8 protein production was induced with 0.1 mM IPTG. To maximize the protein yield, cultures were incubated at 18 °C and 130 rpm overnight. The following day, cells were harvested by centrifugation (4 000 g for 15 min) and used for protein purification immediately.

Purification MBP-GrhJ

For purification of MBP-tagged GrhJ, cells were resuspended in a final volume of 50 mL 20 mM Tris, 400 mM NaCl, 1 mM EDTA, pH 8 (buffer A) + 1 mg mL^{-1} of lysozyme and 1 tablet of protease inhibitor. The suspension was stirred on ice of 30 min, before lysing the cells by ultrasonication (3 cycles; 4 s pulse, 16 s pause, 1 min pulse time). Then, the lysate was cleared by centrifugation (18 000 g for 30 min) and loaded onto one 5 mL MBP-trap column pre-equilibrated with buffer A. Unspecifically bound proteins were removed by washing with 20 CV of buffer A and MBP-GrhJ was eluted with a gradient of buffer A and buffer B (buffer A + 10 mM maltose; 0% B to 100% B within 5 CV). MBP-GrhJ containing fractions identified by SDS-PAGE analysis were pooled, concentrated and supplemented

with 10% glycerol (v/v). Finally, aliquots were prepared, flash frozen in lq. N_2 and stored at -80 °C until further use.

Purification GrhO10, HyalO10 and GB1-HyalJ

For protein purification, freshly harvested cells were resuspended in 50 mM Tris, 300 mM NaCl, 10% glycerol, pH 7.4 (binding buffer) and lysed by ultrasonication (3 s pulse, 2 s, pause, 5 min pulse time). The resulting lysate was cleared by centrifugation (18 000 g for 40 min) and the clear supernatants were loaded onto Ni-NTA FF crude columns pre-equilibrated with binding buffer. Unspecifically bound proteins were removed by extensive washing with wash buffer (10 CV; 50 mM Tris, 300 mM NaCl, 30 mM imidazole, 10% glycerol, pH 7.4) and GrhO10, HyalO10 or GB1-HyalJ was eluted with elution buffer (50 mM Tris, 300 mM NaCl, 500 mM imidazole, 10% glycerol, pH 7.4). GrhO10, HyalO10- or GB1-HyalJ containing fractions identified by SDS-PAGE analysis were pooled and concentrated to 425 µM GrhO10, 460 µM HyalO10 and 580 µM GB1-HyalJ (total protein concentration given; HyalJ constituted ca. 12% of this fraction, i.e. ca. 70 µM based on SDS PAGE analysis) using centripreps. Finally, aliquots were prepared, flash-frozen in liquid nitrogen and stored at -80 °C until further use.

Production and purification GrhO6

Production and purification of GrhO6 was carried out as described previously[1].

Site-directed mutagenesis – cloning of GrhO6 variants

In order to find out whether HyalJ acetylates one of GrhO6's Lys residues, three GrhO6 variants were generated by PCR-based mutagenesis. Mutations were introduced into the pETDuet-GrhO6 wild type construct with forward and reverse primers carrying the desired nucleotide replacements.

Variant	**Primer**	**Nucleotide sequence (5'-3')**
K5M	fwd.	CGGACACC**ATG**GGCACCACCGACACCATC
	rev.	GTGGTGCC**CAT**GGTGTCCGGCATGCTAC
K57M	fwd.	CGCACTCC**ATG**GCGTTCGGGCTGCACGCG
	rev.	CCGAACGC**CAT**GGAGTGCGGGGTGCGCTC
K425M	fwd.	CCCTCCCG**ATG**TTCCTGCGGCGCGGCAAC
	rev.	CGCAGGAA**CAT**CGGGAGGGTCCTGGGCCC

Successful generation of the mutant constructs was confirmed by automated sequencing, prior to transforming the newly produced recombinant plasmids into *E. coli* BL21 (DE3) cells for gene expression. Production and purification of the GrhO6-variants was carried out as described for the wild type protein.

Production and isolation of 7,8-dideoxy-griseorhodin C and chemical oxidation to 7,8-dideoxy-6-oxo-griseorhodin C (6)

Cultivation of the KR7 (GrhO7 knock-out) strain, isolation of **6b** and the subsequent oxidation to **5b** with Dess-Martin-periodinane was carried out as described previously[1].

Cultivation of* Actinoplanes ianthinogenes *and analysis of the accumulating metabolites

A. ianthinogenes (DSM No.: 43864) spores (20 µL) were inoculated into 20 mL GYM medium and cultivated at 28 °C and 180 rpm for 49 h. A 5 mL aliquot of this seed culture was transferred to 200 mL GYM medium and incubation was continued at 30 °C and 200 rpm for 100-200 h.

Then, 2 mL samples were taken and supernatant and pellet were separated by centrifugation (10 min, 18 000 g, 4 °C). Supernatants were subsequently split into portions (4 × 500 µL) and pellets were resuspended in H_2O (500 µL). In both cases samples were acidified with HCl (1 M, 100 µL) before DCM/MeOH (1/1 v/v, 1 mL) was added. The mixtures were shaken vigorously (1 400 rpm, 30 min) and finally phases were separated by centrifugation (5 min, 18 000 g, 4 °C). Organic layers were transferred to fresh reaction tubes and solvents removed *in vacuo*. The residues were redissolved in CH_3CN (200 µL) of which 50 µL were analyzed by HPLC-DAD using a NUCLEODUR 100-5 C18ec column (250 x 10 mm ID, 5 µM, Macherey Nagel).

***Isolation of** 7 **from* Actinoplanes ianthinogenes**

To obtain pure **7** as standard compound for comparison in all activity assays performed in this study, *A. ianthinogenes* was cultured and **7** was isolated as described previously (see compound **12** in Frensch *et al.*[1]). A characterization of the compound was carried out using UPLC-HRMS as well as NMR analysis as shown in Figures S34 (HRMS) and S44-S47 (^{1}H-NMR, ^{13}C-NMR, HSQC and HMBC, respectively) of the same publication.

Cultivation of* Streptomyces puniceus *and analysis of the accumulating metabolites

S. puniceus (DSM No.: 41106) spores (slightly covered pipette tip) were inoculated into 5 mL GYM medium and incubated at 30 °C and 180 rpm for 96 h. A sample (500 µL) of this culture was taken and pellet and culture broth were separated by centrifugation (10 min, 18 000 g, 4 °C). The supernatant was transferred to a fresh tube and the pellet was resuspended in H_2O (100 µL). Both fractions were acidified with HCl (1 M, supernatant: 100 µL; pellet: 20 µL) and extracted with EtOAc (supernatant: 500 µL; pellet: 200 µL). The mixtures were shaken vigorously (1 400 rpm, 15 min) followed by centrifugation (5 min, 18 000 g, 4 °C). The organic layers were transferred to fresh reaction tubes and solvents were removed *in vacuo*. The residues were redissolved in CH_3CN (50 µL) and analyzed by HPLC-DAD using a NUCLEODUR 100-5 C18ec column (250 x 10 mm ID, 5 µM, Macherey Nagel).

Cultivation of* S. albus *pMP31 and analysis of the accumulating metabolites

Cultivation of the *S. albus* pMP31 strain was carried out as described previously[2] and metabolite analysis was carried out as described for the KR42 mutant strain below.

Cultivation of* S. albus *KR42 (Δ*grhJ*) and analysis of accumulating compounds

To analyze the effect of knocking out the acetyltransferase GrhJ on griseorhodin A production *in vivo*, the corresponding *S. albus* strain (KR42) was cultivated in LB-medium supplemented with apramycin (50 µg µL) at 30 °C and 200 rpm for 2-3 days. Then, cells were harvested by centrifugation (4 000 g for 15 min) and the resulting pellet as well as the clear supernatant were acidified with HCl to pH 3 and extracted with EtOAc. Solvents were removed under reduced pressure and residues were redissolved in CH_3CN or CH_3CN:DMSO (1:1) and analyzed by semipreparative HPLC (NUCLEODUR 100-5 C18ec column; 250 x 10 mm ID, 5 µM, Macherey Nagel).

Spontaneous hydrolysis of 7,8-dideoxy-6-oxo-griseorhodin C (5a/b) under assay conditions (in the presence of DTT to generate reducing conditions)

To determine the stability of **5a/b** under assay conditions, 50 µM **5b** and NADPH (0.7 mM) were added to 50 mM Tris, pH 8 (+ 1 mM DTT in a second reaction) and incubated to 30 °C and 750 rpm. Samples were withdrawn after 0, 1, 2, 5, 10 and 30 min and quenched and extracted with EtOAc + 10% FA and the organic layers were analyzed by HPLC-DAD after 30 s of centrifugation at 13 300 g.

Conversion of isolated 7,8-dideoxy-6-oxo-griseorhodin C (5) by GrhO10

To test the activity of GrhO10 on **5a/b**, **5b** was dissolved in DMSO and mixed with 50 mM Tris, pH 7.2 containing 1.5 mM NADH and 1.5 mM NADPH. Then, GrhO10 (40 µM) was added and the reactions were incubated at 30 °C and 750 rpm. Samples were withdrawn after 0, 1, 2, 3, 4, and 5 h and were quenched by the addition of 1.5 eq (v/v) of EtOAc + 10% FA. After 10 min of centrifugation at 13 000 rpm, organic layers were transferred to fresh reaction tubes and dried in the speed-vac. The residues were redissolved in CH_3CN:DMSO (1:1) and analyzed by semipreparative HPLC (NUCLEODUR 100-5 C18ec column; 250 x 10 mm ID, 5 µM, Macherey Nagel).

Reaction cascade with collinone, GrhO5, GrhO1, GrhO6 and GrhO10/HyalO10 – 7,8-dideoxygriseorhodin C (6a/b) formation test

To test the conversion of **5a/b** into **6a/b** by GrhO10/HyalO10 (in the absence of HyalJ), reaction mixtures containing collinone (200 µM), GrhO5 (20 µM), GrhO1 (10 µM), GrhO6 (10 µM) and GrhO10/HyalO10 (45 µM) were prepared. Reactions were started by the addition of NADPH (3 mM) and incubated at 30 °C and 750 rpm. After 10 min of incubation, reactions were quenched by the addition of 2 eq (v/v) of EtOAc + 10% FA and the organic layers were analyzed by HPLC-DAD immediately.

Time course of 7,8-dideoxy-6-oxo-griseorhodin C (5a/b) formation in the presence of HyalJ

To study **5(a)b** formation in the absence and presence of HyalJ, a reaction cascade involving GrhO5 (20 µM), GrhO1 (10 µM), GrhO6 (10 µM), ca. 10 HyalJ µM + AcCoA (300 µM) and **3b** (200 µM) as substrate was set up (control without HyalJ). Reactions were started by the addition of NADPH (3 mM) and incubated at 30 °C and 750 rpm. Samples were withdrawn from the assay mixture after 0, 0.5, 1, 2, 4, 7, 10, and 15 min and quenched with 2 eq (v/v) of EtOAc + 10% FA, each. The 0 min sample was centrifuged at 13 000 g for 30 s and the organic layer was analyzed by HPLC-DAD immediately. All

other samples were flash-frozen in liquid nitrogen right after quenching and thawed and centrifuged only shortly prior to HPLC-DAD analysis to avoid the formation of undesired oxidation products.

Activity test of HyalJ in the absence and presence of AcCoA

To test the effect of AcCoA on HyalJ activity, we set up a reaction cascade involving 20 µM GrhO5, 10 µM GrhO1, 10 µM GrhO6, 10 µM HyalJ (+ 300 µM AcCoA) and using **3b** (200 µM) as substrate. Reactions were started by the addition NADPH (3 mM) and incubated at 30 °C and 750 rpm for 4 min. After quenching with 2 eq (v/v) EtOAc + 10% FA, samples were centrifuged for 30 s and the organic layers were analyzed by HPLC-DAD immediately.

Isolation of AcCoA from HyalJ

To find out, whether HyalJ freshly purified from *E. coli* or stored at -80 °C for several weeks binds AcCoA, 150 µL of a 510/580 µM protein solution containing about 100 µM HyalJ were mixed with 100 µL EtOAc + 10% FA. The organic layer was removed and the aqueous phase was centrifuged at 18 000 g for 10 min. Finally, the clear water phase was analyzed by UPLC-HRMS.

Incubation of HyalJ with 7

Turnover test of **7** with HyalJ and AcCoA. For that, purified **7** was mixed with HyalJ (10 µM) and AcCoA (300 µM) in 50 mM Tris, pH 8 and incubated at 30 °C and 750 rpm for 4-6 min, followed by analytical HPLC as described further below. Under these conditions, no conversion of **7** was observed.

Assay O5/O1/O10 to produce *8* ***for UPLC-HRMS analysis***

To produce compound **8**, 20 µM GrhO5, 10 µM GrhO1, 45 µM HyalO10 and 200 µM **3b** were mixed and incubated at 30 °C for 2 min. Then, NADPH (3 mM) was added, and reactions were incubated at 30 °C and 750 rpm for 10 min. Reactions were quenched and extracted by the addition of 2 eq (v/v) EtOAc + 10% FA and the organic layers were subjected to HPLC-DAD after 30 s of centrifugation at 13 300 g. The peak corresponding to **8** was collected manually and **8** was again extracted from the solvent mixture using EtOAc. Subsequently, the organic phase was transferred to fresh reaction tubes and concentrated in the speed-vac to complete dryness. The residue was redissolved an CH_3CN and **8** was subjected to UPLC-HRMS analysis.

Inhibition assay with inhibitor *8*

To test the effect of **8** on the activity of GrhO6, **8** and **4** were produced in two separate enzymatic assays. Then, 50 µL of the **4a(b)** mixture were combined with varying amounts of the **8** product solution (+50 mM Tris pH 8) and GrhO6 was added to a final concentration of 10 µM. Reactions were incubated at 30 °C and 750 rpm for 10 min, before quenching and extracting them with 200 µL of EtOAc + 10% FA. Samples were flash-frozen in liquid nitrogen and thawed and centrifuged (13 000 g for 30 s) shortly before analyzing the organic layers by HPLC-DAD.

Activity GrhO6 variants

Activity of the GrhO6-variants was tested both in the absence and presence of HyalJ. Therefore, 200 µM **3b**, 20 µM GrhO5, 10 µM GrhO1 (+ 10 µM HyalJ) and 10 µM of a GrhO6-variant were mixed and incubated at 30 °C and 750 rpm for 2 min. Then, NADPH (3 mM) was added and reactions were incubated at 30 °C and 750 rpm for 4 min. Reactions were quenched and extracted with 2 eq (v/v) of EtOAc + 10% FA and flash-frozen in liquid nitrogen. Finally, samples were thawed and centrifuged (13,300 g for 30 s) shortly prior to analysis of the organic layers by HPLC-DAD.

Homology modeling

Homology models of GrhO10 and HyalJ were generated using the SWISS-MODEL server[3, 4] and the Phyre[2] server[5], respectively. For GrhO10, the crystal structure of the C17/C19-ketoreductase ARX21 (PDB-ID, 5thq) was used as a template, as this protein exhibited the highest sequence identity and coverage (60% identity on amino acid sequence level and 100% coverage) of all crystallized proteins with GrhO10. For HyalJ, the crystal structure of a GCN5-related acetyltransferase from clavulanic acid biosynthesis (PDB-ID, 2wpw) sharing 23% sequence identity (95% coverage) with HyalJ was selected as template. Despite the not so high sequence similarity, a confidence of 100% was predicted for the model, which can be explained by the fact that GCN5-related acetyltransferases generally share high structural but low sequence similarity.

Multiple sequence alignment

Structure based multiple sequence alignments of ARX21 and several GrhO10-homologs were generated using the online server T-coffee[6]. The alignment results were exported in PHYLIP format and visualized in SeaView[7]. Final color editing was carried out in PowerPoint.

Analytical gel filtration general

To determine the biologically active oligomeric state of the proteins used in this study, analytical gelfiltration using a Superdex 200 GL 10/300 column pre-equilibrated with 50 mM Tris, 300 mM NaCl, 10% glycerol, pH 7.4 was used. Molecular weights of the proteins eluting in the main peak was calculated based on a previously generated calibration curve.

HPLC analyses general

Turnover assays were analyzed by HPLC using an Agilent 1100 chromatographic system (Technologies), equipped with a VP NUCLEODUR 100-5 C18ec (250 × 4.6 mm ID, 5 µM, Macherey-Nagel) or a semipreparative VP NUCLEODUR 100-5 C18ec (250 x 10 mM ID, 5 µM, Macherey-Nagel) column coupled with a UNIVERSAL RP guard column (4 × 3 mm ID, Macherey-Nagel). The analytical column was pre-equilibrated with ddH_2O + 0.1% TFA (solution A) and acetonitrile + 0.1% TFA (solution B) 90:10 (v/v) and samples were analyzed at a flow rate of 1.5 mL min^{-1} using the following gradient: 10% B (0-1 min), 10-100% B (1-15 min), 100% B (15-20 min), 10% B (20-25 min). The semipreparative column was pre-equilibrated with ddH_2O + 0.1 % TFA (solution A) and acetonitrile + 0.1% TFA (solution B) 98:2 (v/v) and samples were analyzed at a flow rate of 5 mL min^{-1} using the following gradient: 2% B (0-1 min), 2-100% B (1-15 min), 100% B (15-20 min), 100-2% B (20-21 min),

2% B (21-23 min). Considering the strong absorption of all analyzed compounds in the visible-light region, absorption changes at 254, 350, and 500 nm were selected to be monitored by the diode array detector connected to the chromatographic system.

UPLC-MS analysis general

LC-HRMS analyses were performed using a Waters Acquity UPLC H class system coupled with a diode array detector (Waters). For all analyses, an analytical RP C18 column (Acquity UPLC HSS T3, 100Å, 1.8 µm, 2.1 x 100 mm; Waters) was used, pre-equilibrated with 10 mM AmAc pH 6.8 (solution A1) and acetonitrile (solution B1) 98:2 (v/v) for analysis of aqueous samples or with ddH_2O + 0.1% FA (solution A2) and acetonitrile + 0.1% FA (solution B2) 98:2 (v/v) for **8** characterization. Samples were analyzed at a flow rate of 0.2 mL min-1 using the following gradients: 2% B1/B2 (0-1 min), 2% to 60% B1/B2 (1-11 min), 60% B1/B2 (11-11.5 min), 60% to 2 % B1/B2 (11.5-11.6 min), 2% B1/B2 (11.6-14 min). Samples were analyzed in MS positive mode with a capillary voltage of 3.0 kV, 100 °C source temperature, 300 °C desolvation gas temperature and 600 L h^{-1} N_2 desolvation gas flow and a capillary voltage of 1.5 kV, 120 °C source temperature, 300 °C desolvation gas temperature and 500 L h^{-1} N_2 desolvation gas flow for MS negative mode.

2. Supplementary Figures and Tables

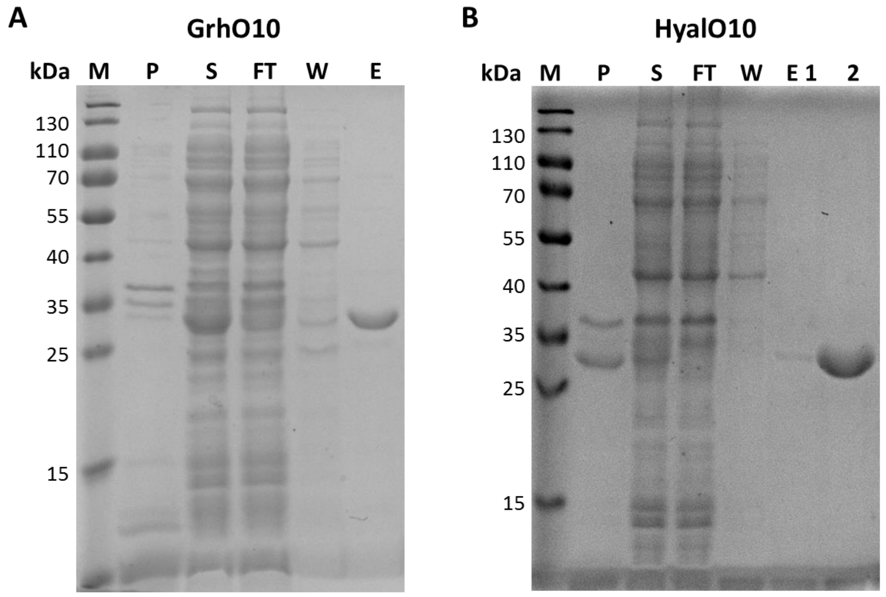

Figure S1: **SDS-PAGEs showing the different fractions collected in the course of the affinity purification of GrhO10 (A) and HyalO10 (B).** Page ruler prestained protein ladder (**M**), cell-pellet after lysis (**P**), cleared cell lysate (**S**), column flow-through (**FT**), wash fraction (**W**), elution fractions (**E, E1, 2**).

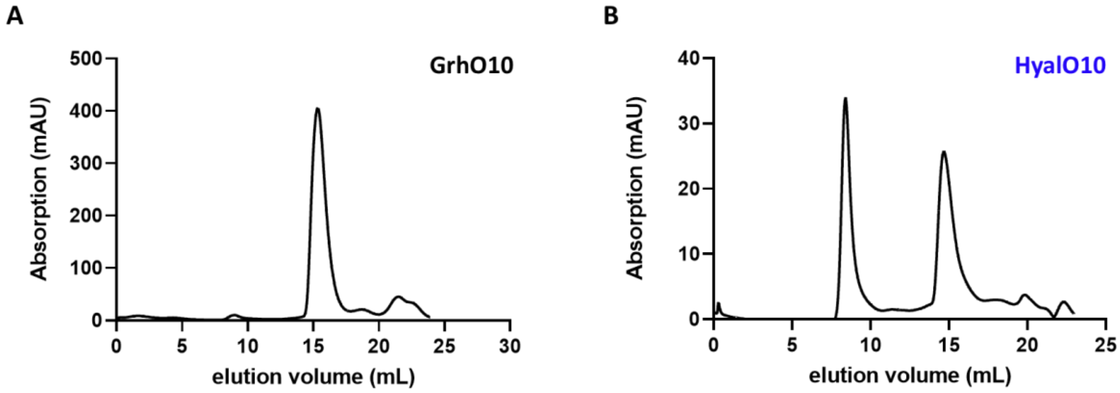

Figure S2: Analytical gelfiltration of GrhO10 and HyalO10. Analytical size-exclusion was performed for both proteins using a Superdex 200 GL 10/300 column pre-equilibrated with 50 mM Tris, 300 mM NaCl, 10% glycerol, pH 7.4. GrhO10 eluted as a clean single peak at around 15 mL, indicating that it forms dimers in solution. For HyalO10 also a peak corresponding to dimeric enzyme was detected at 15 mL, however, also a major aggregate peak around 8-8.5 min was observed.

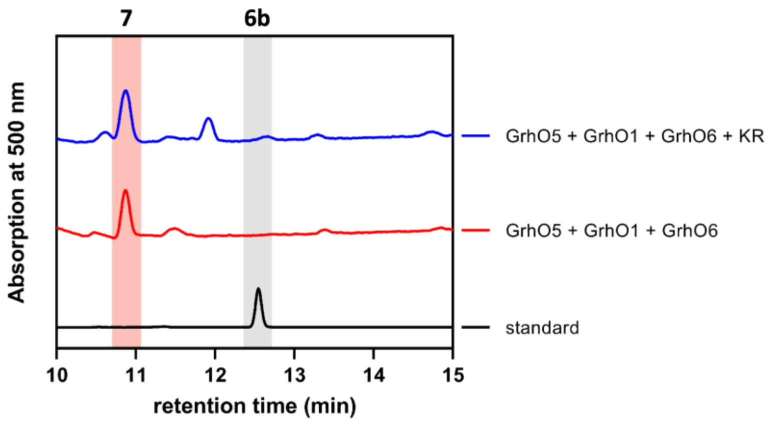

Figure S3: HPLC-chromatograms showing the results of a turnover assay with 3b**, GrhO5, GrhO1, GrhO6 and the ketoreductase (KR).** To test the activity of the KR, a reaction cascade with **3b**, NADPH, GrhO5, GrhO1, GrhO6 and the KR (control without KR) was used. Interestingly, in either case, **6(a)b** (**6b** standard, *black* line; highlighted in *grey*) formation could not be observed. Both in the reaction with as well as without the KR, **7** (highlighted in *light red*) was the main accumulating metabolite. The peak at 12 min in the *blue* trace is inhibitor **8**.

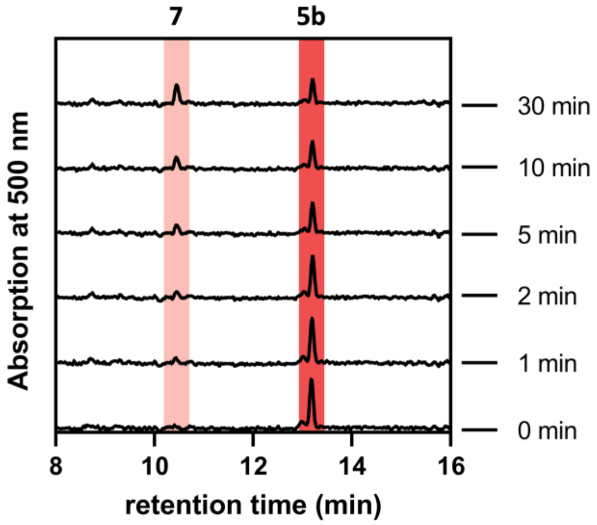

Figure S4: Time-dependent spontaneous hydrolysis of 5b **under assay/reducing conditions. 5b** was dissolved in DMSO and added to 50 mM Tris, 300 mM NaCl, 10% glycerol (50 µM) additionally containing 0.7 mM NADPH and 1 mM DTT to generate reducing conditions. Reactions were incubated at 30 °C and 750 rpm and samples were withdrawn after 0, 1, 2, 5, 10 and 30 min and analyzed by HPLC-DAD. As indicated by the time course graph, **5b** (*dark red*) is slowly converted into **7** (*light red*).

```
ARX21   MELELGLRGK KALVTGGSRG VGRGVVLALA RAGVDVFTCY REESDASASL ARELKQLGGD HHALRADLAD  70
GrhO10  --MELGLQGK KLLVTGGSRG IGRGVVLAAA RAGADVLTCY VNDSEHVESL RRELKALGGD HEVVRADVGD  68
HyalO10 --MELALHGK KVLITGGTRG IGRGIVLAAA QAGADVLTCH RQDGAAVDSL VQALKETEGD HHVIRADVGD  68
RubG    --MELALHGR KLLITGGTRG IGRGIVLAAA RAGADVLTCY RQESEAVDSL TAALKETAGD HHVIRADVGD  68

ARX21   PKQIAELFQE VG--R-RFGT LDVVVNNAGV ISHVPYAELP VAEWQRIVDV NLTGAHLVIQ HAIPLLGDKG 137
GrhO10  LDQVDAL--- VGWARERYGK LDGIVNNAGV ISHIPFAELA PDEWSRVLDT NLTAAYRVIQ QSLPLLGSGA 135
HyalO10 LDQVDRLVNE AK---DRFGR LDGVVNNAGV ISHVPFAELP AAEWSRILDT NLTASYRVIQ QALPLLGAGS 135
RubG    TAEADRLVGE AK---DRFGR LDGVVNNAGV ISHIPFGELP ADEWSRILDT NLTAAYRIIH QALPLLGTGS 135

ARX21   SVISIGSKSS EVGIPLRAHY TATKHALRGL TRSLAKEYGR SGLRFNVLAL GVVETEELHA LPDDERAE-M 206
GrhO10  SVVNIGSRGA AAGIPLRAHY TAAKAAMIGL TRSLAKELGG KGIRVNVVAP GVIETEAFDT MPA-ERAEGL 204
HyalO10 SVINIGSRGA AAGIPLRAHY TAAKAALIGL TRSLAKELGP QGIRVNVVAP GVIETEAFDD MPADRAAG-L 204
RubG    SVVNIGSRGA AAGIPLRAHY TAAKAAMIGL TRSLAKELGP KGIRVNVVAP GVIETEAFAD MPA-ERAAGL 204

ARX21   TKFYSTKTAL GRLGTPDEVA GAVAWLASDL SRYVTGATIH VDGGIS                          252
GrhO10  RAMYAQKTAL ARLGRVDELA APVLFLLSDL AAYVTGETLN VDGGI-                          249
HyalO10 RATYAQKTAL ARLGTVDELA GPVLFLLSDL STYVTGETVN VDGGI-                          249
RubG    RATYSQKTAL ARLGTVEELA GPVLFLLSDA AAYITGETLN VDGGI-                          249
```

Figure S5: Multiple sequence alignment of the ketoreductases GrhO10 (griseorhodin A biosynthesis), HyalO10 (hyaluromycin biosynthesis), RubG (rubromycin biosynthesis) and the structurally characterized C17/C19 ketoreductase ARX21 (polyphenol biosynthesis). When compared with structurally characterized ketoreductases, GrhO10, HyalO10 and RubG all share the highest sequence identity and coverage with ARX21 (60% identity, 100% coverage), a C17/C19 ketoreductase from pentangular polyphenol biosynthesis. Conserved sequence motifs unique to C19 ketoreductases are highlighted in bold. The proposed substrate binding and catalytic residues are additionally colored in *red* and *blue*, respectively.

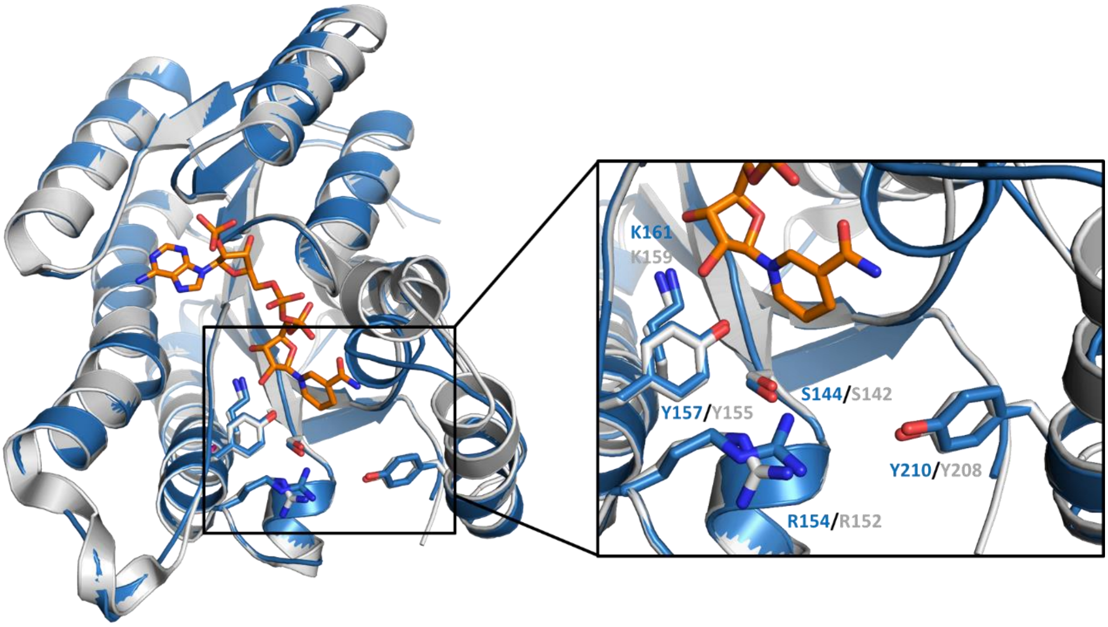

Figure S6: Overlay of the crystal structure of the C17/C19 ketoreductase ARX21 (*grey*) with a homology model generated for GrhO10 (*blue*). Comparison of the homology model of GrhO10 (*blue*) with the crystal structure of ARX21 (*grey*; PDB-ID, 5thq) reveals a virtually identical overall fold as well as conserved active site residues. Both the catalytic triad proposed for ARX21 consisting of Y144, Y157, and K161, as well as the binding residues (R154 and Y210) are found in GrhO10 as well (Y142, Y155 and K159 and R154 and Y208, respectively). All proposed key residues are shown as sticks (ARX21, *grey*; GrhO10, *blue*). The $NADP^+$ observed in the crystal structure of ARX21 is displayed in *orange*.

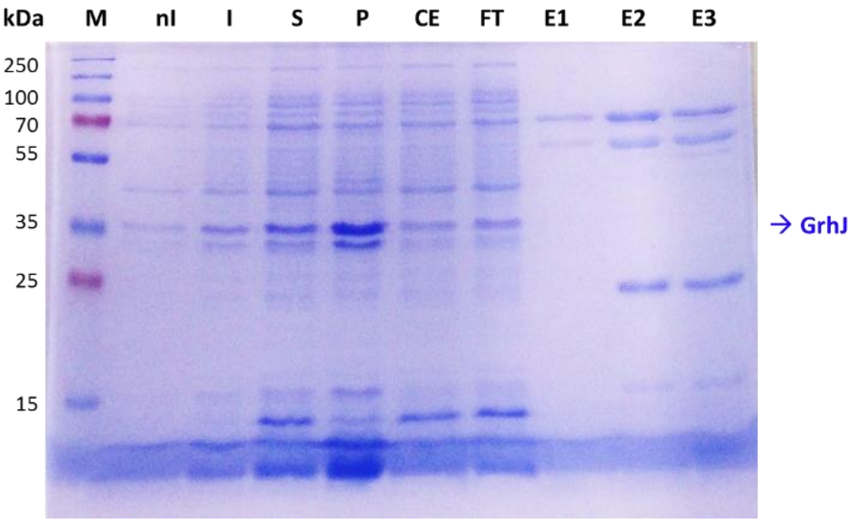

Figure S7: SDS-PAGE showing the different fractions collected in the course of the affinity purification of His$_6$-GrhJ. Lane 1, Page ruler prestained plus protein ladder (**M**); lane 2, cell extract pre-induction (**nI**); lane 3, cell-extract post induction (**I**); lane 4, cleared cell lysate (**S**); lane 5, cell-pellet after lysis (**P**); lane 6, cell extract (**CE**); lane 7, column flow-through (**FT**); lane 8-10, elution fractions (**E1-E3**).

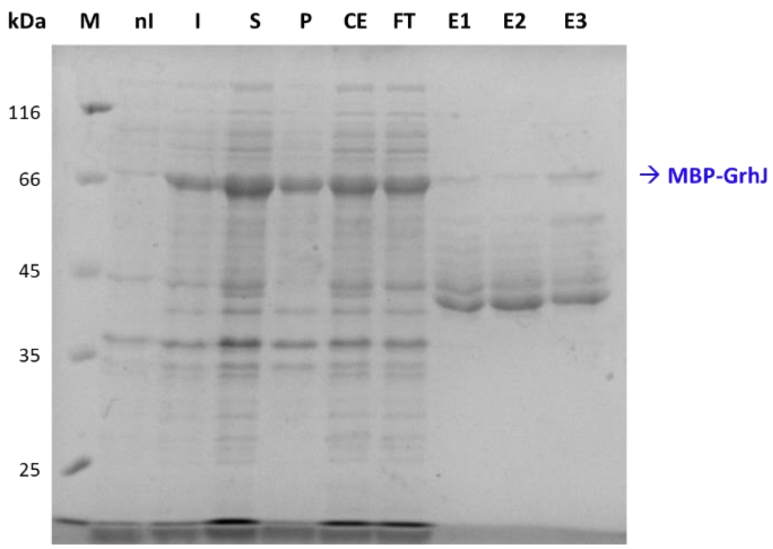

Figure S8: SDS-PAGE showing the different fractions collected in the course of the affinity purification of MBP-GrhJ. Lane 1, Page ruler unstained protein ladder (**M**); lane 2, cell extract pre-induction (**nI**); lane 3, cell-extract post induction (**I**); lane 4, cleared cell lysate (**S**); lane 5, cell-pellet after lysis (**P**); lane 6, cell extract (**CE**); lane 7, column flow-through (**FT**); lane 8-10, elution fractions (**E1-E3**).

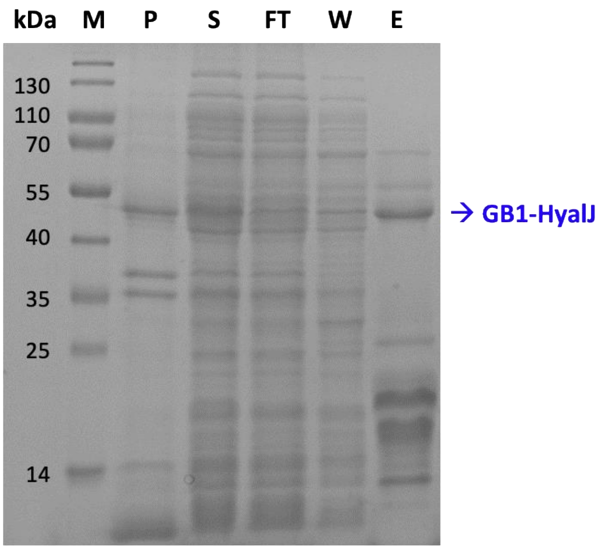

Figure S9: **SDS-PAGE showing the different fractions collected in the course of the affinity purification of GB1-HyalJ.** Lane 1, Page ruler prestained protein ladder (**M**); lane 2, cell-pellet after lysis (**P**); lane 3, cleared cell lysate (**S**); lane 4, column flow-through (**FT**); lane 5, wash fraction (**W**); lane 5, elution fraction (**E**).

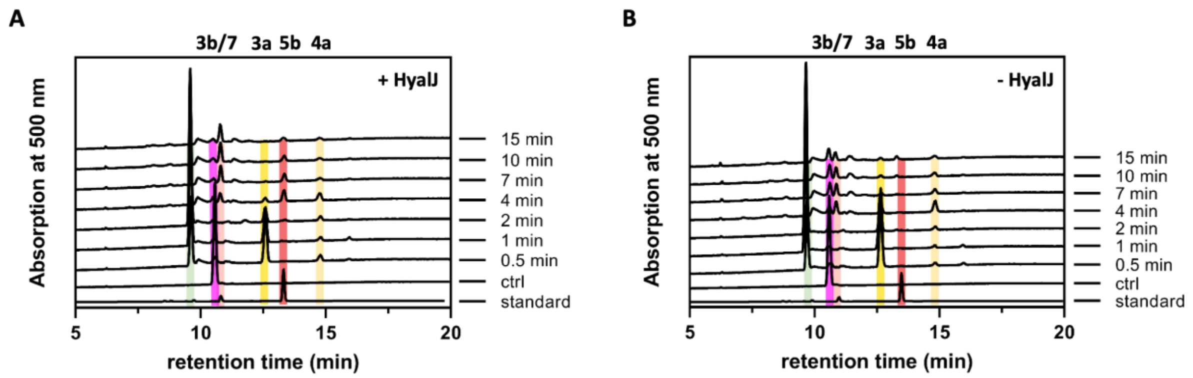

Figure S10: Uncropped chromatograms showing the time course for the formation of 5b **in a reaction cascade containing** 3b**, NADPH, GrhO5, GrhO1, GrhO6 (and HyalJ). A**, In the reaction with HyalJ, after the initial reduction of **3b** (ctrl, *pink* line – 10.5 min) to **3a** (*yellow* line – 12.5 min) by GrhO5, **4a** (*pale yellow* line – 14.7 min) is formed by the combined action of GrhO5 and GrhO1. **4a** is then further converted into **5b** (*dark red* line – 13.3 min) by GrhO6 together with HyalJ. At the same time, the shunt product **7** (light red line – 10.7 min) is generated either by GrhO6 not interacting with HyalJ or via spontaneous hydrolysis of **5b**. **B**, In the reaction without HyalJ, after the initial reduction of **3b** (ctrl, *pink* line – 10.5 min) to **3a** (*yellow* line – 12.5 min) by GrhO5, **4a** (*pale yellow* line – 14.7 min) is formed by the combined action of GrhO5 and GrhO1, too. However, **4a** is then directly further converted into **7** (*light red* line – 10.7 min) by GrhO6. In this case, no **5b** (standard, *dark red* line – 13.3 min) is detected.

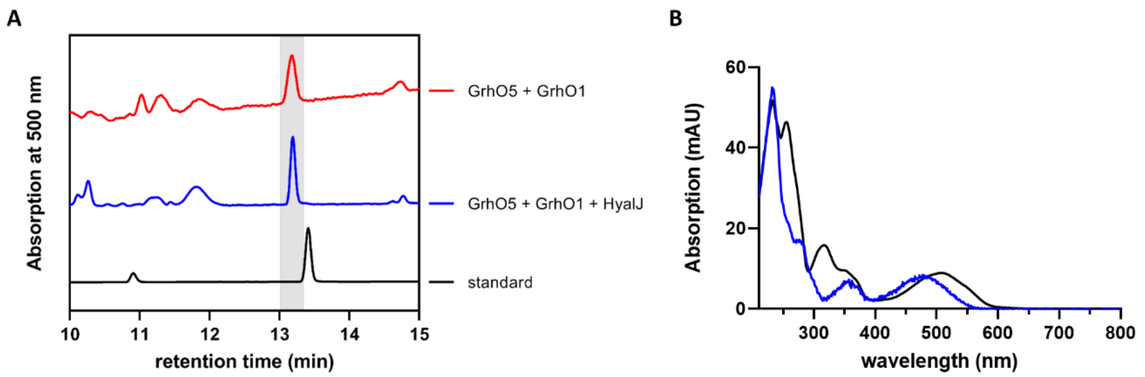

Figure S11: Effect of HyalJ on 3a/b **turnover by GrhO5 and GrhO1.** To find out whether HyalJ is able to convert **4a(b)**, turnover assays with **3b**, NADPH, GrhO5 and GrhO1 were performed in the absence (*red* line) and presence (*blue* line) of HyalJ. In both assays, the identical reaction product (**A**, *red* and *blue* line), but not **5a/b** (**A**, **5b** standard, *black* line) was found – see also difference in UV-visible absorption characteristics of the formed compounds (**B**, *blue* line) compared with the standard (**B**, *black* line), suggesting that HyalJ does not modify their initial reaction product **4a(b)**. Note, the product detected in this assay does only accumulate upon prolonged incubation with GrhO1 in the absence of the downstream enzyme GrhO6.

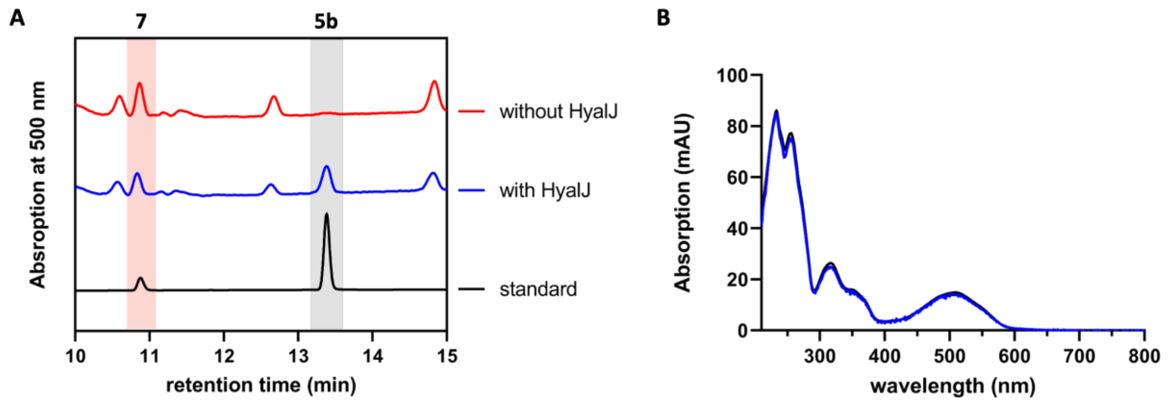

Figure S12: Effect of HyalJ on 3a/b **turnover by GrhO5, GrhO1 and GrhO6.** To test the effect of HyalJ on **5a/b** formation, turnover assays with **3b**, NADPH, GrhO5, GrhO1 and GrhO6 were performed in the absence and presence of HyalJ. While only shunt product **7** was detected in the absence of HyalJ, a new peak with an identical retention time (**A**) and absorption characteristics (**B**; standard – *black* line, from assay – *blue* line) compared with a **5b** standard (*black* line) was found in the presence of the acetyltransferase.

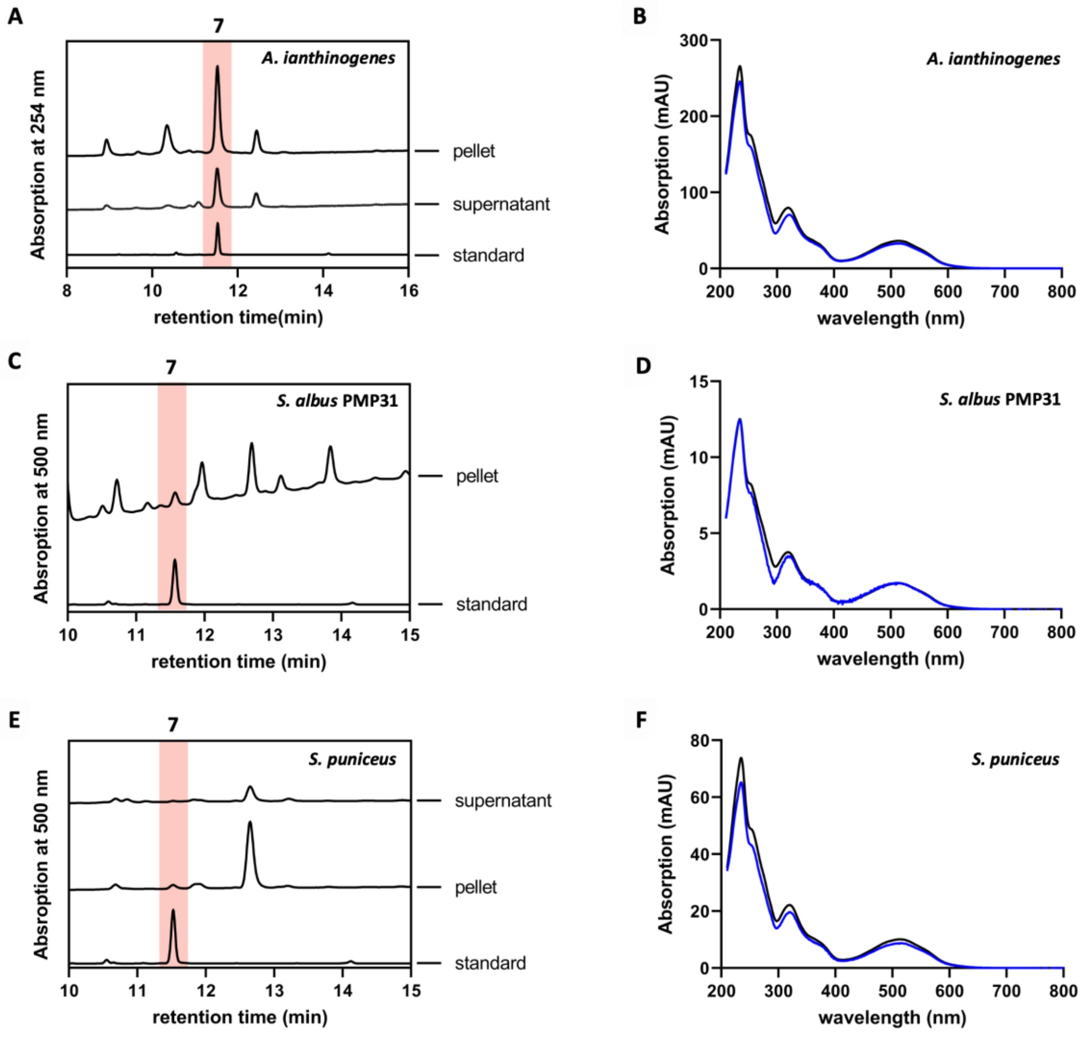

Figure S13: Chromatographic analysis of the cells and the culture supernatant of *A. ianthinogenes*, *S. albus* PMP31 and *S. puniceus* compared with a 7 **standard.** Upon extraction with EtOAc + 10% FA and subsequent HPLC-DAD analysis, shunt product **7** was detected in the culture supernatant of *A. ianthinogenes* and *S. puniceus* as well as in the cell pellet of all three cultured strains. In these cases, both an identical retention time (**A, C, E**) as well as the same absorption characteristics (**B, D, F** - standard, *black* line; pellet, *blue* line) were found compared with the standard compound, indicating that **7** accumulates even in wild type strains capable of producing different mature rubromycin polyketides.

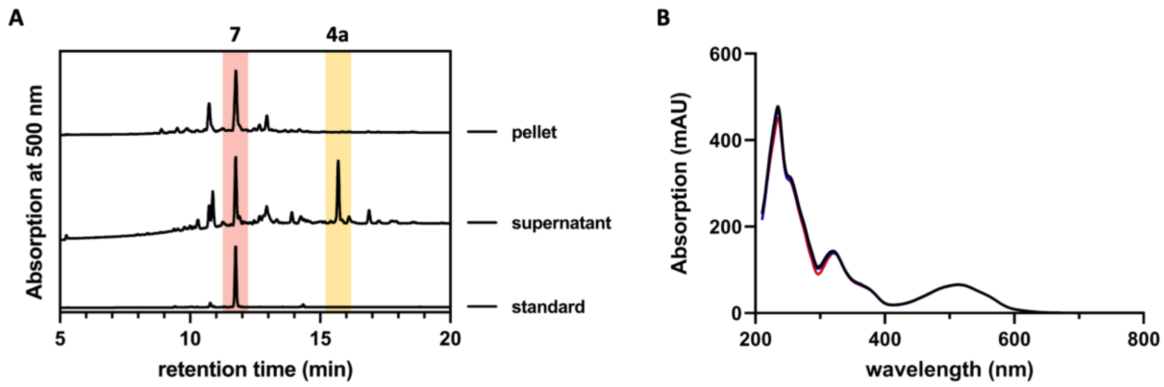

Figure S14: Chromatographic analysis of the cells and the culture supernatant of the Δ*grhJ* strain KR42 compared with a 7 **standard.** Upon extraction with EtOAc + 10% FA and subsequent HPLC-DAD analysis, major amounts of the shunt product **7** were detected in the culture supernatant as well as in the cell pellet - in both cases an identical retention time (**A**) as well as the same absorption characteristics (**B** - standard, *black* line; supernatant, *blue* line; pellet, *red* line) were found compared with the standard compound - indicating that the acetyltransferase GrhJ plays a role in the formation of **5**.

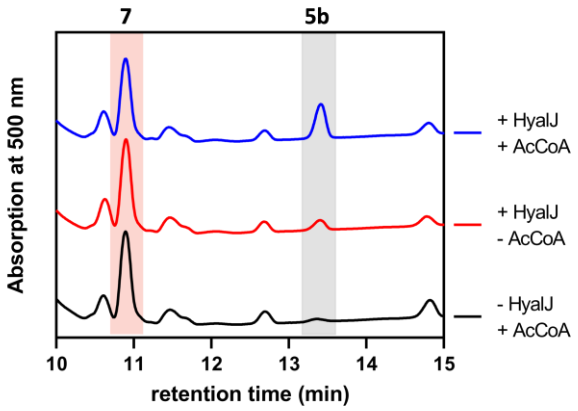

Figure S15: **Effect of free acetyl-CoA (AcCoA) on (dihydro-)7,8-dideoxy-6-oxo-griseorhodin C (**5a/b**) formation.** To test the effect of acetyl-CoA on HyalJ activity, turnover assays with **3b**, NADPH, GrhO5, GrhO1, GrhO6 and HyalJ were performed in the absence and presence of free AcCoA. In line with its proposed function, the addition of acetyl-CoA strongly increased the enzymatic activity of HyalJ resulting in elevated **5a/b**-levels (highlighted in *grey*) in this sample.

Table S1: Effect of AcCoA on 7,8-dideoxy-6-oxo-griseorhodin C formation by the acetyltransferase HyalJ.

Sample	Peak area (AUC at 500 nm)
+ Hyal J / - AcCoA	49 ± 13
+ HyalJ / + AcCoA	107 ± 3
- HyalJ / + AcCoA	n.d.

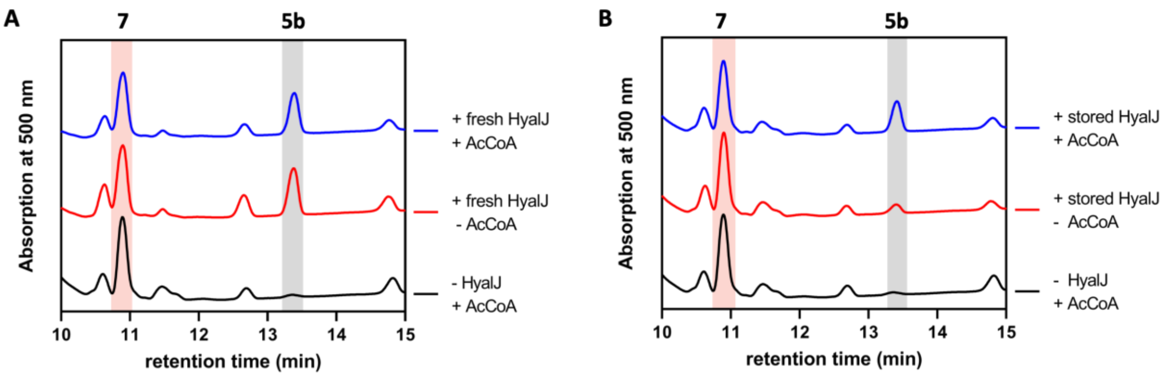

Figure S16: Effect of acetyl-CoA (AcCoA) on 5a/b **formation.** To test the effect of AcCoA on HyalJ activity, turnover assays with **3b**, NADPH, GrhO5, GrhO1, GrhO6 and HyalJ were performed in the absence and presence of free AcCoA and analyzed by HPLC-DAD. While hardly any influence of free acetyl-CoA on **5b** formation could be observed with freshly purified HyalJ (**A**), this effect became rather pronounced when then protein had already gone through several freezing-thawing cycles (“stored HyalJ”; **B**).

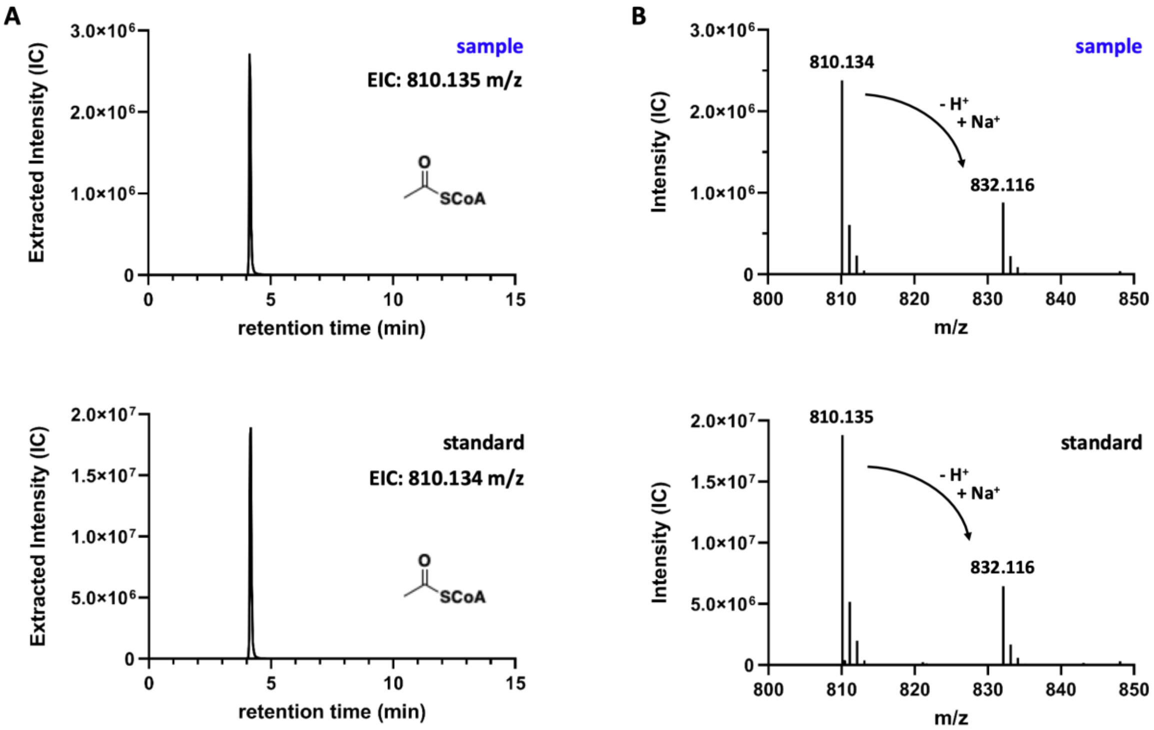

Figure S17: **HyalJ is produced and purified as holo-enzyme with acetyl-CoA bound in the active site.** To find out, whether HyalJ is co-purified with acetyl-CoA, freshly purified HyalJ was sacrificed with EtOAc + 10% FA. After removal of the organic phase, the water phase was centrifuged to remove protein particles and the aqueous layer was analyzed by UPLC-HRMS. In line with the enzymatic assays, acetyl-CoA could be detected in the water phase, confirming that HyalJ is produced and purified as holo-enzyme. **A**, Extracted ion chromatograms (positive ion mode) corresponding to acetyl-CoA ($[M+H]^+$, 810.135 m/z) detected in the sample (*top*) as well as in a pure standard (*bottom*). **B**, Mass spectra corresponding to the peaks shown in panel A. As indicated, besides the mother ion ($[M+H]^+$, 810.135 m/z), Na^+-adducts were also detected.

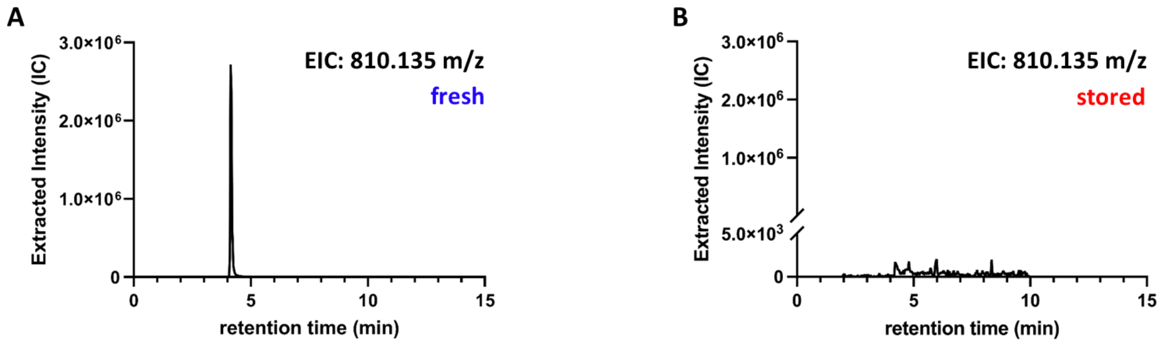

Figure S18: Acetyl-CoA content in fresh and stored HyalJ. To confirm that HyalJ "inactivation" upon long-term storage/after several freezing-thawing cycles results from acetyl-CoA degradation, "poorly active" HyalJ was sacrificed with EtOAc + 10% FA. After removal of the organic phase, the water phase was centrifuged to remove protein particles and the aqueous layer was analyzed by UPLC-HRMS. In line with the enzymatic assays, in contrast to the freshly purified enzyme (**A**) no acetyl-CoA could be detected in the water phase of the stored enzyme (**B**), confirming that HyalJ "inactivation" is the result of acetyl-CoA degradation. This is also in line with the fact that addition of free acetyl-CoA to "inactivated" HyalJ can restore its enzymatic activity.

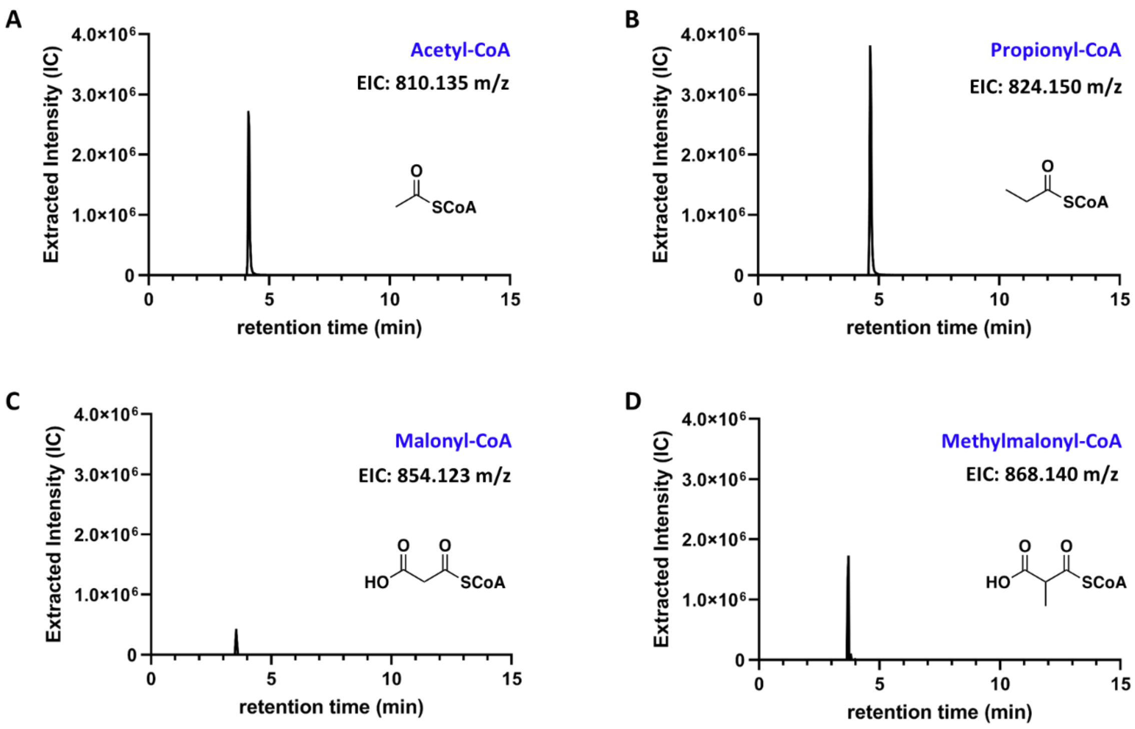

Figure S19: CoA derivatives identified to be co-purified with HyalJ. To find out, whether HyalJ is co-purified with acetyl-CoA or any other CoA-derivative, freshly purified HyalJ was sacrificed with EtOAc + 10% FA. After removal of the organic phase, the water phase was centrifuged to remove protein particles and the aqueous layer was analyzed by UPLC-HRMS. Interestingly, not only acetyl-CoA but also propionyl-CoA, malonyl-CoA and methylmalonyl-CoA could be detected. While malonyl-CoA was only present in small amounts, the signal intensity for propionyl-CoA and methylmalonyl-CoA was in the same range as for acetyl-CoA. **A-D**, Extracted ion chromatograms (positive ion mode) corresponding to acetyl-CoA (**A**; $[M+H]^+$, 810.135 m/z), propionyl-CoA (**B**; $[M+H]^+$, 824.150 m/z), malonyl-CoA (**C**; $[M+H]^+$, 854.123 m/z) and methylmalonyl-CoA (**D**; $[M+H]^+$, 868.140 m/z), respectively.

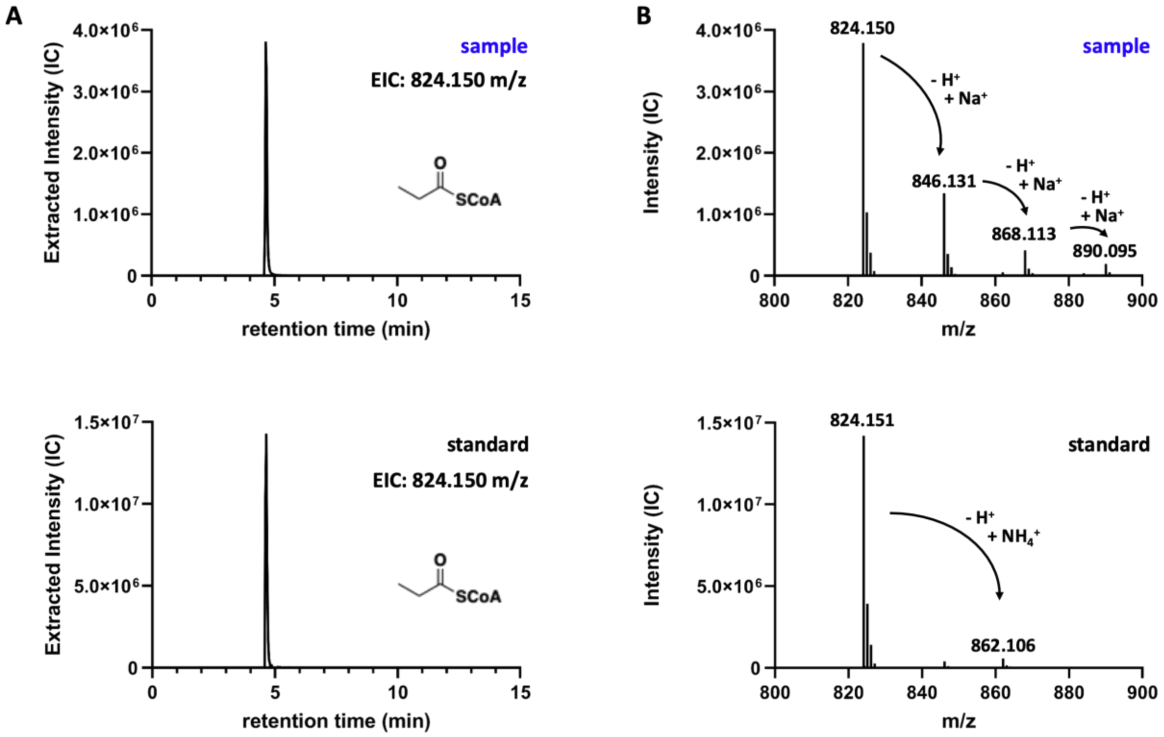

Figure S20: HyalJ is purified with bound propionyl-CoA. UPLC-HRMS analysis of the metabolites present in the water phase after sacrificing freshly purified HyalJ with EtOAc + 10% FA revealed that HyalJ is not only co-purified with acetyl-CoA, but also with propionyl-CoA. **A**, Extracted ion chromatograms (positive ion mode) corresponding to propionyl-CoA ($[M+H]^+$, 824.150 m/z) detected in the sample (top) as well as in a pure standard (bottom). **B**, Mass spectra corresponding to the peaks shown in panel A. As indicated, besides the mother ion ($[M+H]^+$, 824.150 m/z) also several Na^+-adducts were detected in the sample, whereas only minor amounts of an NH_4^+-adduct were observed in the standard.

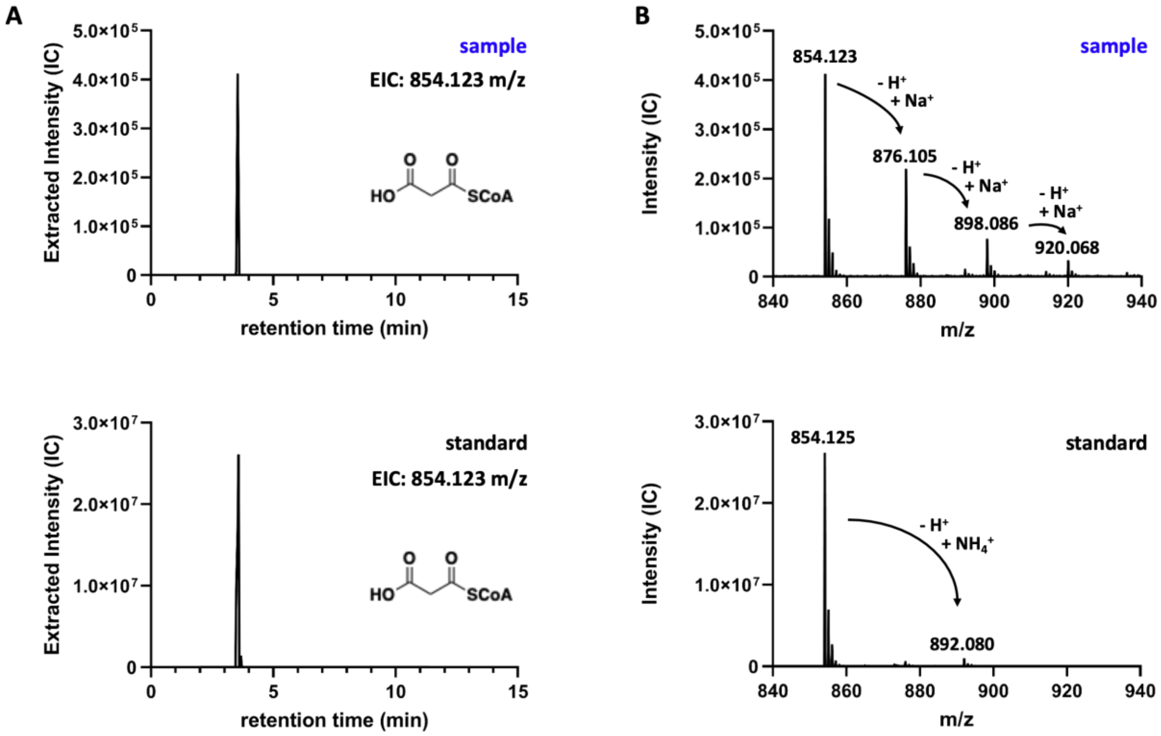

Figure S21: HyalJ is purified with bound malonyl-CoA. UPLC-HRMS analysis of the metabolites present in the water phase after sacrificing freshly purified HyalJ with EtOAc + 10% FA revealed that HyalJ is not only co-purified with acetyl-CoA, but also with malonyl-CoA. **A**, Extracted ion chromatograms (positive ion mode) corresponding to malonyl-CoA ($[M+H]^+$, 854.123 m/z) detected in the sample (top) as well as in a pure standard (bottom). **B**, Mass spectra corresponding to the peaks shown in panel A. As indicated, besides the mother ion ($[M+H]^+$, 854.123 m/z) also several Na^+-adducts were detected in the sample, whereas only minor amounts of an NH_4^+-adduct were observed in the standard.

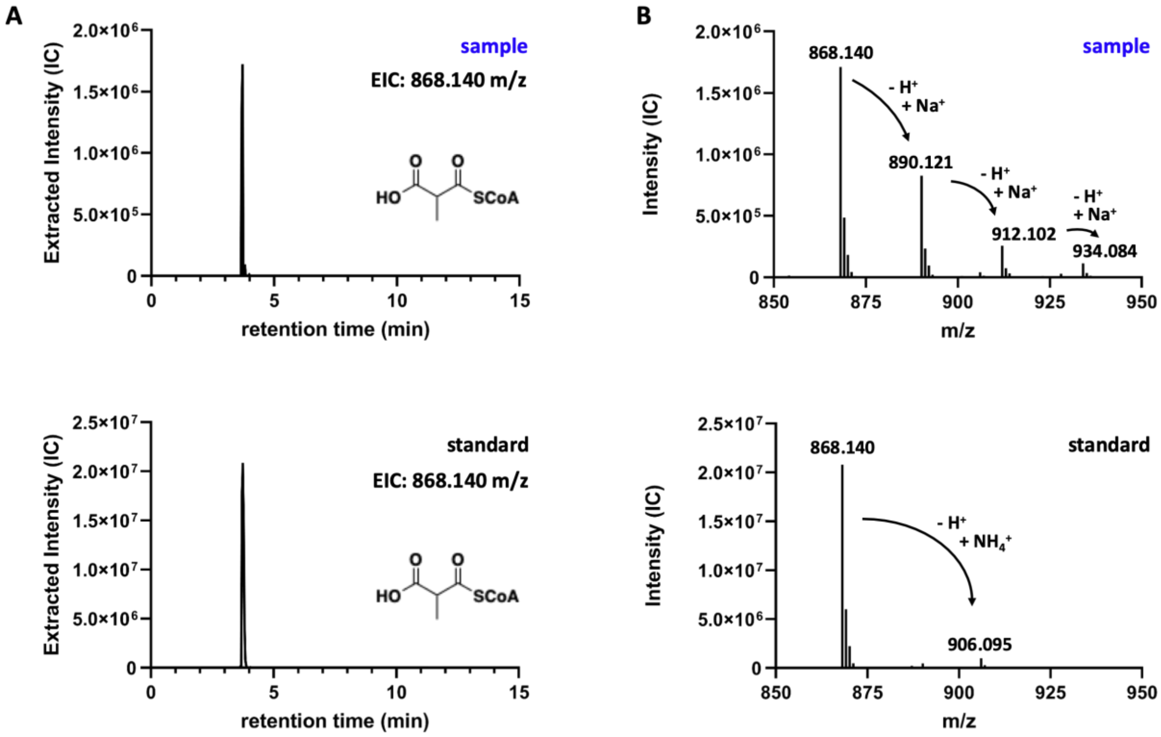

Figure S22: HyalJ is purified with bound methylmalonyl-CoA. UPLC-HRMS analysis of the metabolites present in the water phase after sacrificing freshly purified HyalJ with EtOAc + 10% FA revealed that HyalJ is not only co-purified with acetyl-CoA, but also with methylmalonyl-CoA. **A**, Extracted ion chromatograms (positive ion mode) corresponding to methylmalonyl-CoA ($[M+H]^+$, 868.140 m/z) detected in the sample (top) as well as in a pure standard (bottom). **B**, Mass spectra corresponding to the peaks shown in panel A. As indicated, besides the mother ion ($[M+H]^+$, 868.140 m/z) also several Na^+-adducts were detected in the sample, whereas only minor amounts of an NH_4^+-adduct were observed in the standard.

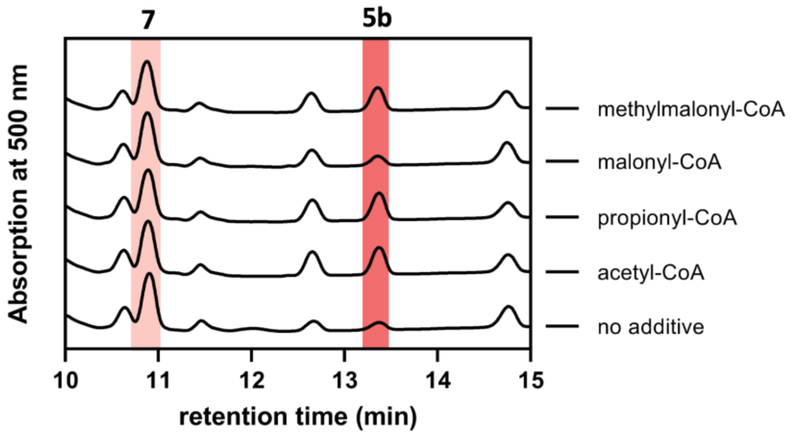

Figure S23: HyalJ activity in the presence of different CoA-derivatives. To find out, whether HyalJ is only active with acetyl-CoA or also with the other CoA-derivatives detected upon UPLC-HRMS analysis of freshly purified enzyme, activity assays with “stored” HyalJ were carried out (as described above) in the presence of acetyl-CoA (as control), propionyl-CoA, malonyl-CoA and methylmalonyl-CoA. Interestingly, **5b** (highlighted in *dark red*) formation could be boosted to similar levels as with acetyl-CoA both by the addition of propionyl-CoA and methylmalonyl-CoA. If, however, malonyl-CoA was provided to the acetyltransferase only the basal activity of enzyme (without the addition of any CoA-derivative) was detectable.

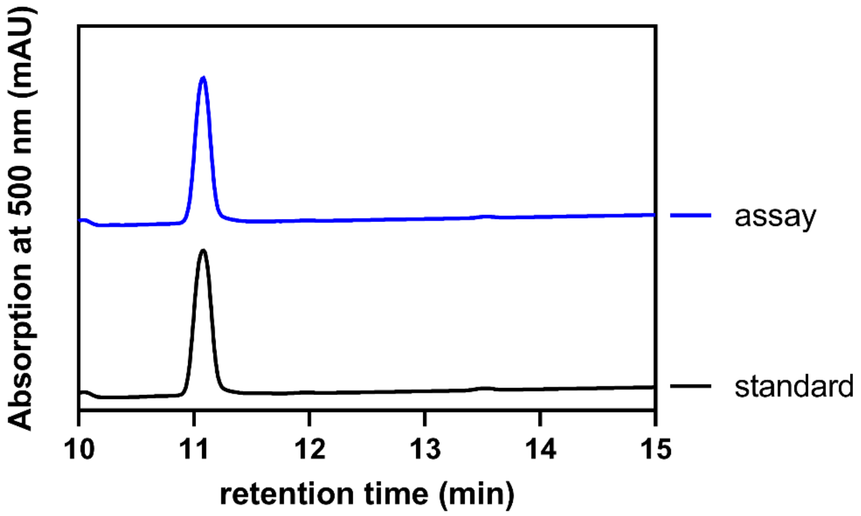

Figure S24: Turnover test of 7 **with HyalJ and AcCoA.** To test the activity of HyalJ on **7**, purified **7** was mixed with HyalJ (10 µM) and AcCoA (300 µM) and incubated. Under these conditions, no conversion of **7** into **5** (standard, *black* line) was observed.

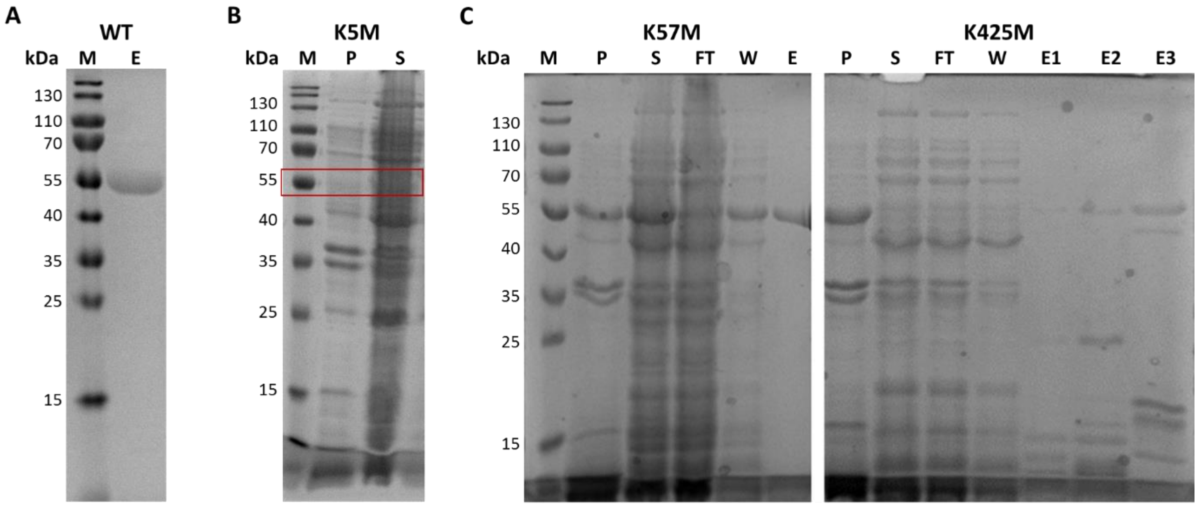

Figure S25: **SDS-PAGE analysis of GrhO6 purified via Ni-NTA-chromatography (A), overproduction analysis of GrhO6-K5M (B) and SDS-PAGE showing the different fractions collected in the course of the affinity purification of GrhO6-K57M and GrhO6-K425M (C).** Page ruler prestained protein ladder (**M**), cell-pellet after lysis (**P**), cleared cell lysate (**S**), column flow-through (**FT**), wash fraction (**W**), elution fraction (**E**). While GrhO6-WT (A) and GrhO6-K57M (C, *left*) could be produced in decent amounts, only little GrhO6-K425M (C, *right*) could be obtained from the same amount of culture. The K5M variant (B) was even found not to be overproduced at all.

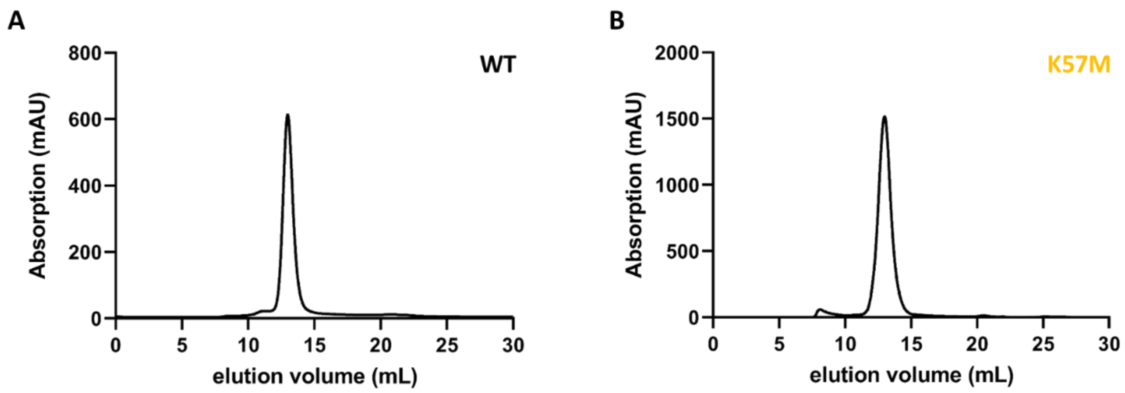

Figure S26: Analytical gelfiltration of GrhO6-WT and GrhO6-K57M. Analytical size-exclusion was performed for both proteins using a Superdex 200 GL 10/300 column pre-equilibrated with 50 mM Tris, 300 mM NaCl, 10% glycerol, pH 7.4. Both proteins eluted as clean single peaks at around 13 mL, indicating that they form dimers in solution.

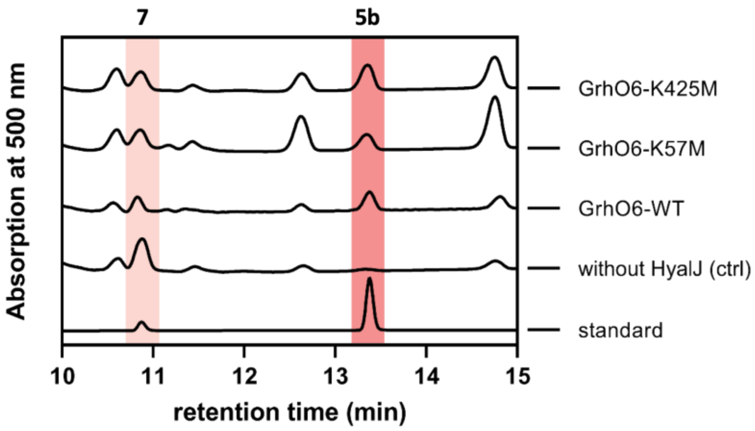

Figure S27: Effect of two different lysine substitutions in GrhO6 on 5b **formation.** To test the effect of two different lysine substitutions on GrhO6 activity and on its ability to form **5** in the presence of HyalJ, reaction mixtures containing **3b**, NADPH, GrhO5, GrhO1, HyalJ (+ AcCoA) and one of the GrhO6 variants were incubated at 30 °C and 750 rpm for 4 min. Then, all reactions were quenched and extracted with EtOAc + 10% FA and the organic layers were analyzed by HPLC-DAD. Both GrhO6-K57M and -K425M like the wild type protein were able to mediate **5** (highlighted in *dark red*) formation, indicating that K57 and K524 are not the sites of acetylation used by HyalJ.

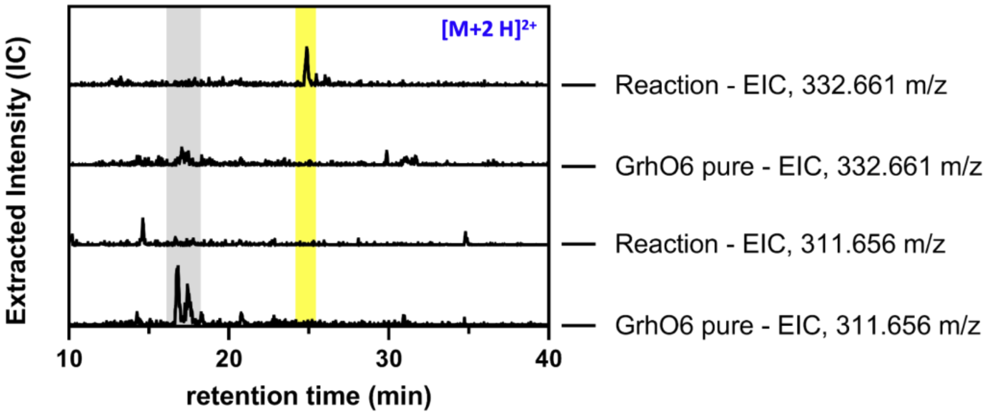

Figure S28: HRMS-analysis of pure GrhO6 and GrhO6 after turnover of 4a/b **in the presence of HyalJ.** Pure GrhO6 and GrhO6 from the reaction mixture were purified by SDS-PAGE and subsequently in-gel digested with an *N*-Asp protease. Afterwards, extracted protein fragments were applied to UPLC-HRMS analysis, which revealed that reacted GrhO6 is acetylated at K5, as a $[M+2H]^{2+}$-fragment of 332.661 m/z corresponding to DT**K**(Ac)TT was detectable in this sample (highlighted in *yellow*) that was not observed in the control containing pure GrhO6. At the same time, the unmodified counterpart ($[M+2H]^{2+}$, 311.656 m/z) of the same fragment could well be detected in the control, (highlighted in *grey*) but not in the sample with reacted GrhO6.

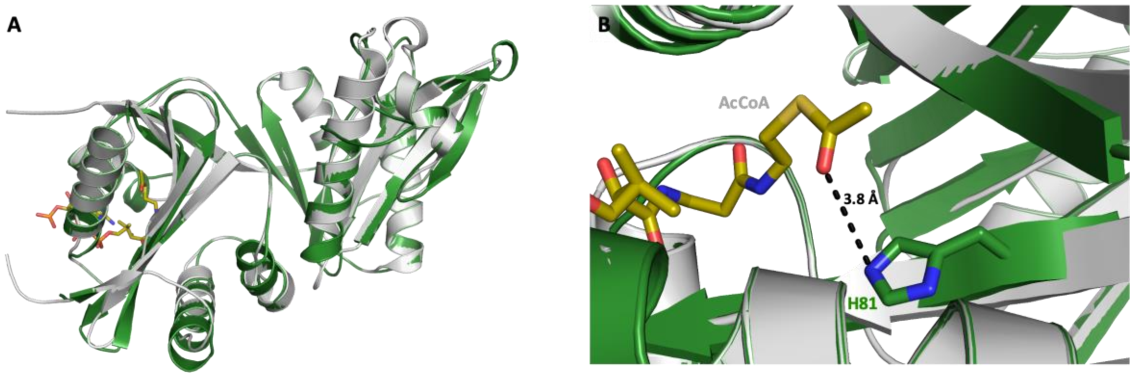

Figure S29: Comparison of a homology model of the GCN5-related N-acetyltransferase (GNAT) HyalJ (green) with the crystal structure of a GNAT protein from clavulanic acid biosynthesis (CBG, grey). A, Overall predicted structure of HyalJ (*green*) overlaid with the crystal structure of CBG (*grey*; PDB-ID, 2wpw). **B**, Close-up view of the putative AcCoA binding site as well as active site of HyalJ (*green*). His81, which is in hydrogen bonding distance to the carbonyl group of the thioester, may be the catalytic base in HyalJ or may be responsible for the stabilization of the transition state.

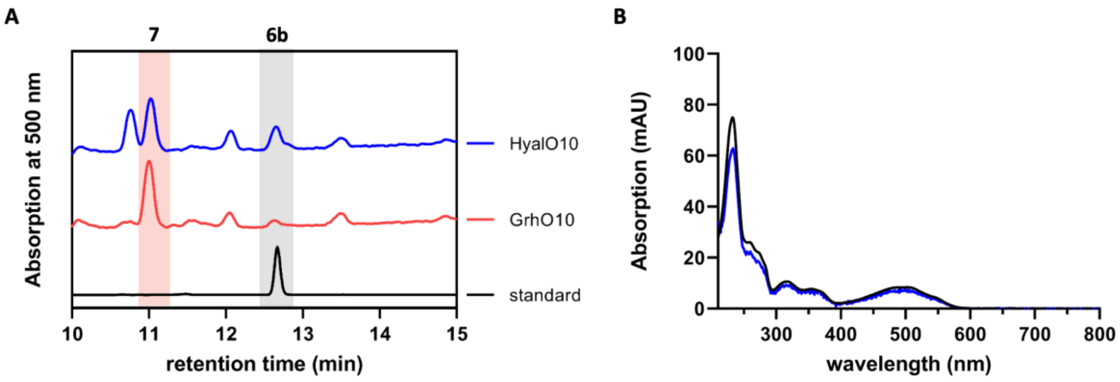

Figure S30: 6a/b **formation by GrhO10 and HyalO10. 6** formation was tested in a reaction mixture with **3b**, NADPH, GrhO5, GrhO1, GrhO6, HyalJ (+AcCoA) and one of the ketoreductases (GrhO10 or HyalO10). **A**, Comparison of the HPLC-traces corresponding to the assays with GrhO10 (*red*) or HyalO10 (*blue*). In both assays a peak with an identical retention time like the **6b** standard (*black* line) was detected (highlighted in *grey*). **B**, UV-visible absorption spectra corresponding to the peaks at 12.7 min – **6b** standard (*black* line) and reaction with HyalO10 (*blue* line). For the same peak in the sample with GrhO10 due to the low signal intensity no absorption spectrum can be shown.

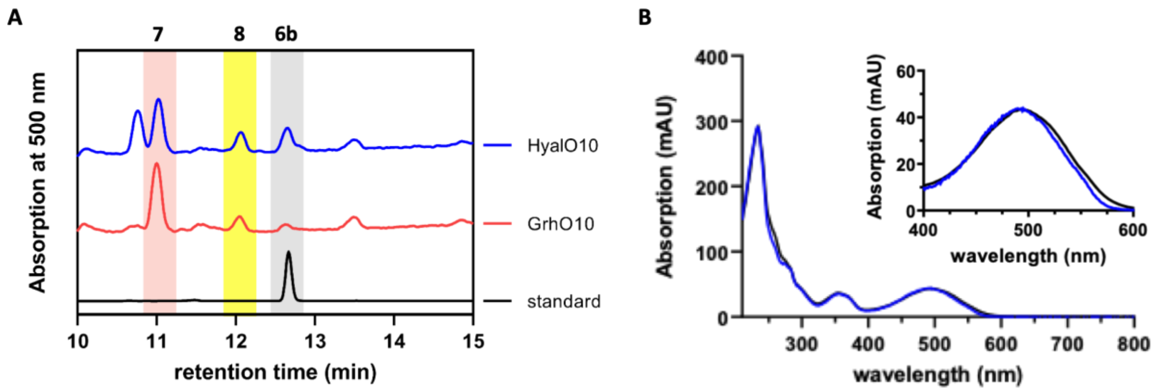

Figure S31: (Dihydro-)7,8-dideoxygriseorhodin C (6a/b**) formation by GrhO10 and HyalO10 and identification of the lenticulone derivative (**8**)**. Formation of **6a/b** was tested in a reaction mixture with **3b**, NADPH, GrhO5, GrhO1, GrhO6, HyalJ and one of the ketoreductases (GrhO10 or HyalO10). **A**, Comparison of the HPLC-traces corresponding to the assays with GrhO10 (*red*) or HyalO10 (*blue*). In both assays, a peak with an identical retention time like **6b** standard (*black* line) was detected (highlighted in *grey*). In addition, a second new peak at around 12.0 min (highlighted in *yellow*) was observed, corresponding to the **4b** derivative with a C12-hydroxyl group (**8**) resulting from GrhO10/HyalO10-catalyzed keto reduction. **B**, UV-visible absorption spectra of **8** and **4b**. The high similarity of the UV-visible absorption spectra corroborates that compound **8** derives from **4b** and has virtually the same chromophore. *Inset*, Zoom of the UV-visible absorption spectrum shown in panel B between 400 and 600 nm.

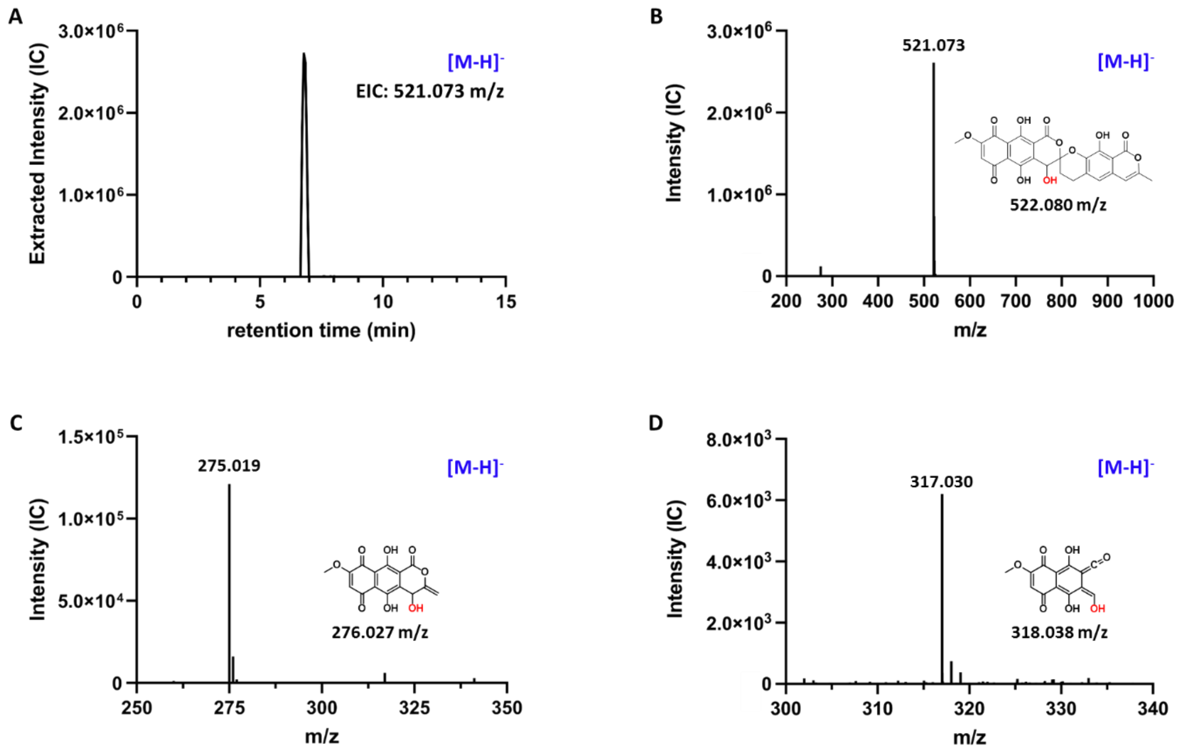

Figure S32: UPLC-HRMS analysis of compound 8. **A**, Extracted ion chromatogram corresponding to a mass of 521.073 m/z detected in negative ion mode. **B**, Mass spectrum corresponding to compound **8** obtained in negative ion mode. **C**, Zoom of the mass spectrum shown in panel B in the range from 250-350 m/z. The structure of the fragment corresponding to the mass of 275.019 m/z (neg. mode) is shown next to the signal. **D**, Zoom of the mass spectrum shown in panel B in the range from 300-340 m/z. The structure of the fragment corresponding to the mass of 317.030 m/z (neg. mode) is shown next to the signal. NOTE: The compound is clearly not reduced at ring A, as ring A reduction strongly affects the UV-visible absorption characteristics of the metabolites (see Frensch *et al.* [1] for spectral comparison).

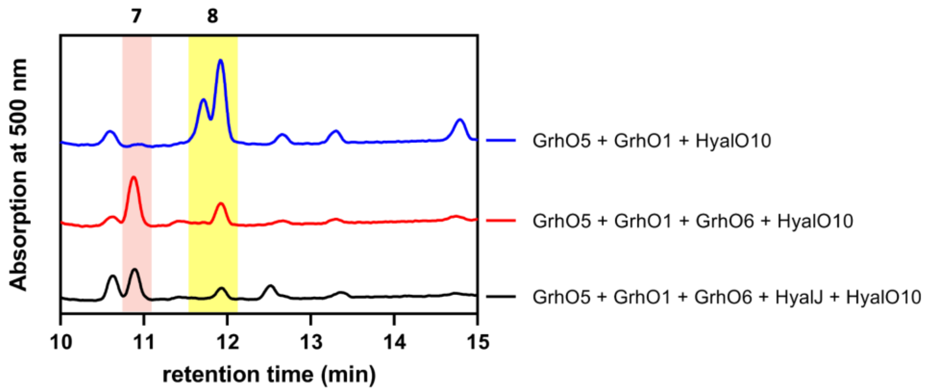

Figure S33: 8 **formation by the ketoreductase HyalO10.** To find out which metabolite is converted into **8** (highlighted in *yellow*) by the ketoreductases GrhO10 and HyalO10, different enzymatic assays were performed. *Red* line, To test the requirement of HyalJ for **8** formation, a reaction mixture with **3**, NADPH, GrhO5, GrhO1, GrhO6 and HyalO10 was prepared and incubated at 30 °C and 750 rpm for 10 min. The reaction was quenched and extracted with EtOAc + 10% FA and the organic layer was analyzed by HPLC-DAD. Since even higher amounts of **8** were produced under these reaction conditions, compared to the original set up, HyalJ is not involved in **8** formation. *Blue* line, To further analyze the need of GrhO6 for **8** production, the same assay was repeated, however without the addition of GrhO6. In this case, **8** became the major reaction product, indicating that GrhO6 and HyalO10 act on the same substrate – **8** is therefore generated from **4a(b)**.

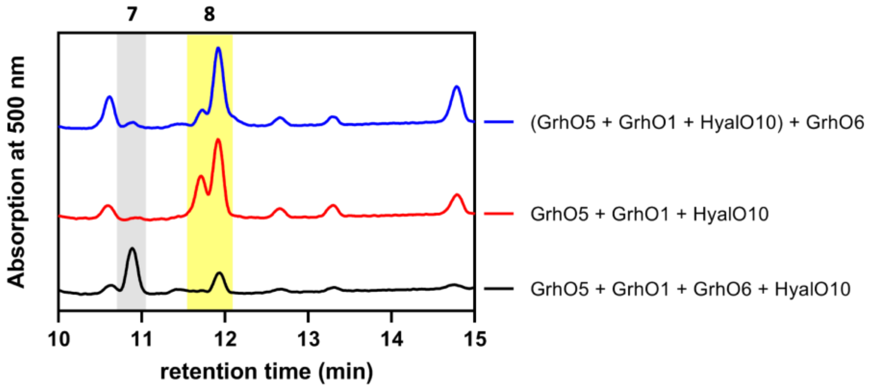

Figure S34: Conversion of 8 **by GrhO6.** To find out, whether GrhO6 can convert **8** (highlighted in *yellow*), GrhO6 was mixed with *in situ* produced **8** and the reactions were incubated at 30 °C and 750 rpm for 10 min. After quenching and extracting with EtOAc + 10% FA, the organic layers were analyzed by HPLC-DAD. As reflected by the chromatogram highlighted in *blue*, GrhO6 is not capable of converting **8** (the slightly different shape of the peak(s) corresponding to **8** probably is the result of tautomerism as the ratio of the two peaks both corresponding to **8** was different each time the assay was repeated).

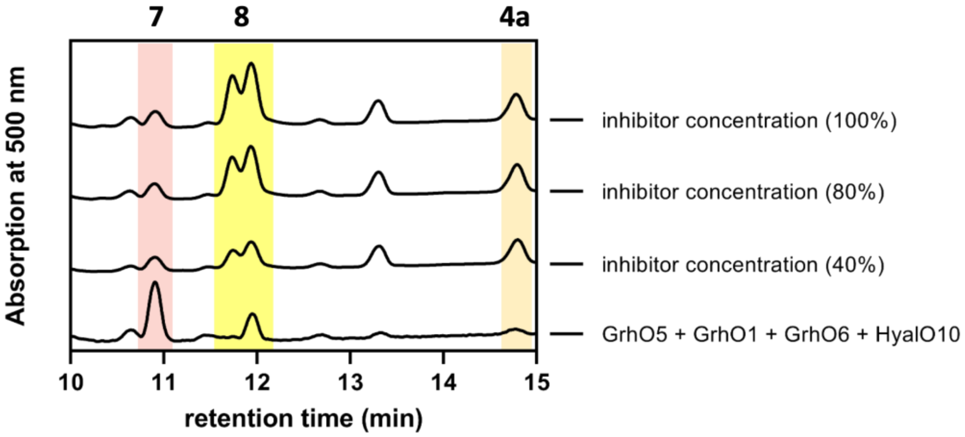

Figure S35: Inhibition of GrhO6 by compound 8**.** To test the inhibitory effect of **8** on GrhO6, **8** was produced in a reaction cascade with **3b**, NADPH, GrhO5, GrhO1 and HyalO10. Then, different amounts of this mixture were added to *in situ* produced **4a/b** before addition of GrhO6. Interestingly, the presence of only minor amounts of **8** (40% inhibitor concentration) were sufficient to almost completely prevent **4a/b** conversion into **7** by GrhO6 compared to a control reaction lacking **8**, in which GrhO6 was added to the reaction mixture with GrhO5, GrhO1 and HyalO10 from the very beginning (bottom line). Compound **8**, the GrhO6 produced **7**, and **4a/b** are highlighted in *yellow*, *light red* and *pale orange*, respectively.

3. References

1. B. Frensch, T. Lechtenberg, M. Kather, Z. Yunt, M. Betschart, B. Kammerer, S. Ludeke, M. Muller, J. Piel and R. Teufel, *Nat. Commun.*, 2021, **12**, 1431.
2. Z. Yunt, K. Reinhardt, A. Li, M. Engeser, H. M. Dahse, M. Gutschow, T. Bruhn, G. Bringmann and J. Piel, *J. Am. Chem. Soc.*, 2009, **131**, 2297-2305.
3. S. Bienert, A. Waterhouse, T. A. de Beer, G. Tauriello, G. Studer, L. Bordoli and T. Schwede, *Nucleic Acids Res.*, 2017, **45**, D313-D319.
4. A. Waterhouse, M. Bertoni, S. Bienert, G. Studer, G. Tauriello, R. Gumienny, F. T. Heer, T. A. P. de Beer, C. Rempfer, L. Bordoli, R. Lepore and T. Schwede, *Nucleic Acids Res.*, 2018, **46**, W296-W303.
5. L. A. Kelley, S. Mezulis, C. M. Yates, M. N. Wass and M. J. Sternberg, *Nat Protoc*, 2015, **10**, 845-858.
6. C. Notredame, D. G. Higgins and J. Heringa, *J Mol Biol*, 2000, **302**, 205-217.
7. M. Gouy, S. Guindon and O. Gascuel, *Mol Biol Evol*, 2010, **27**, 221-224.
